# Addressing proteolytic efficiency in enzymatic degradation therapy for celiac disease

**DOI:** 10.1038/srep30980

**Published:** 2016-08-02

**Authors:** Martial Rey, Menglin Yang, Linda Lee, Ye Zhang, Joey G. Sheff, Christoph W. Sensen, Hynek Mrazek, Petr Halada, Petr Man, Justin L McCarville, Elena F. Verdu, David C. Schriemer

**Affiliations:** 1 Department of Biochemistry and Molecular Biology and the Southern Alberta Cancer Research Institute, University of Calgary, Calgary, Alberta, Canada; 2 Structural Mass Spectrometry and Proteomics Unit, Institut Pasteur, CNRS UMR 3528, Paris, France; 3 Graz University of Technology, Institute of Molecular Biotechnology, Graz, Austria; 4 Institute of Microbiology, Academy of Sciences of the Czech Republic, and Department of Biochemistry, Faculty of Science, Charles University in Prague, Prague, Czech Republic; 5 Farncombe Family Digestive Health Research Institute, McMaster University, Hamilton, ON, Canada

## Abstract

Celiac disease is triggered by partially digested gluten proteins. Enzyme therapies that complete protein digestion *in vivo* could support a gluten-free diet, but the barrier to completeness is high. Current options require enzyme amounts on the same order as the protein meal itself. In this study, we evaluated proteolytic components of the carnivorous pitcher plant (*Nepenthes* spp.) for use in this context. Remarkably low doses enhance gliadin solubilization rates, and degrade gliadin slurries within the pH and temporal constraints of human gastric digestion. Potencies in excess of 1200:1 (substrate-to-enzyme) are achieved. Digestion generates small peptides through nepenthesin and *neprosin*, the latter a novel enzyme defining a previously-unknown class of prolyl endoprotease. The digests also exhibit reduced TG2 conversion rates in the immunogenic regions of gliadin, providing a twin mechanism for evading T-cell recognition. When sensitized and dosed with enzyme-treated gliadin, NOD/DQ8 mice did not show intestinal inflammation, when compared to mice challenged with only pepsin-treated gliadin. The low enzyme load needed for effective digestion suggests that gluten detoxification can be achieved in a meal setting, using metered dosing based on meal size. We demonstrate this by showing efficient antigen processing at total substrate-to-enzyme ratios exceeding 12,000:1.

Celiac disease (CD) is a chronic autoimmune enteropathy, with a defined environmental trigger in the form of gluten proteins consumed from common grain products^1^. Genetically susceptible individuals respond to gluten intake with the presentation of villous atrophy of the small intestine, crypt hyperplasia and mucosal inflammation. The associated intestinal and extra-intestinal symptoms of the disease strongly reduce quality of life.

Wheat gluten consists of plant storage proteins called prolamins, which are deposited in the endosperm of the developing cereal grain. It is comprised mostly of gliadin and glutenin, and when mixed with water it generates molecular networks that impart useful viscoelastic properties to flour dough^2^. These prolamins have high levels of proline (15%) and glutamine (35%)^3^, in sequence combinations that render them resistant to complete proteolysis by gastric enzymes^4^. The sequences convey poor overall digestion kinetics, generating peptides of 30–40 amino acid residues in length that resist further digestion by both intestinal exo- and endoproteases^5^. A fraction of these products, primarily from α and γ-gliadin, have affinity for human leukocyte antigen (HLA) DQ2 and DQ8, which are MHC class II molecules associated with over 90% of CD patients^6^,^7^,^8^. The peptides are large enough to span multiple antigenic regions, and present glutamine residues for enzymatic deamidation in the celiac mucosa. The inflammatory response is significantly amplified by this deamidation, as HLA affinity is increased by the conversion of glutamine to glutamate^9^,^10^,^11^.

A gluten-free diet (GFD) is the primary and obvious treatment option for CD patients. However, maintaining a truly gluten-free status is both difficult and costly^12^,^13^. Compliance issues and hidden gluten contamination produce a constant low-level stimulation that remains the norm for a very large fraction of CD patients. Supplemental or even alternative treatment options are desirable but the bar is necessarily high^14^,^15^. Alternative therapies must be safe, and at least as effective as a GFD in reducing both inflammation and pain. A variety of strategies are being considered based on our improved understanding of disease mechanism^16^,^17^, but successful alternatives to a GFD have yet to emerge^18^.

One compelling approach involves enzyme supplementation of the GI tract, to avoid the induction of immune responses by reducing peptide size in the key antigenic regions that bind to HLA and stimulate inflammation^5^. A small number of candidates have been tested for such purposes, mostly involving prolyl endoproteases (PEPs) or prolyl oligopeptidases (POPs). Two options in advanced testing are AN-PEP^19^,^20^,^21^, a prolyl endoprotease from *Aspergillus niger*, and ALV003^22^, a combination of a POP from *Sphingomonas capsulate* and a glutamine-targeting cysteine endoprotease^23^. To be effective, enzyme supplements should function within the pH constraints of the stomach and both AN-PEP and ALV003 appear to do so. They are well tolerated, and clinical data are emerging that demonstrate the enzymes can attenuate intestinal injury^21^,^24^,^25^.

However, the complexity of the total protein load in a typical meal should define the supplementation strategy and the appropriate dosage. Food products such as dairy, red meat and fish contain proteins with high levels of proline^26^, and will increase the substrate load for therapies dependent solely upon prolyl endoproteases. For example, proline is the most abundant amino acid in beta-casein, at roughly the same fractional level as gliadin. A cheese sandwich would contribute not only 1.6 g of gliadin but also 1.8 g of beta casein, doubling the substrate load for PEP-based therapies. Total protein is therefore a safer measure of substrate load, estimated at 50 g/day, when considering enzyme-based gluten detoxification therapies as an alternative to a GFD. Achieving efficacy at a reasonable dosage remains an obstacle to replacing a GFD. Current candidates appear best suited to supplementing a GFD in situations of limited consumption^21^.

We set 50 g/day as the target in a search for enzymes with improved gluten detoxification potential. One source with considerable promise is the *Nepenthes* genus of carnivorous plants^27^,^28^,^29^. Native to the Old World tropics, plants in this genus have captivated botanists and ecologist for centuries, yet they have only recently inspired investigations into the biochemical and physicochemical elements of their behavior^30^,^31^,^32^,^33^,^34^,^35^,^36^,^37^. Commonly called monkey cups, these plants capture and digest prey from the environment to produce a major fraction of their nutrient requirements, with the aid of a viscoelastic digestive fluid. The fluid sits in the bottom of pit-fall traps that resemble pitchers, in what could be described as a single-stage gastrointestinal (GI) tract. In this study, we demonstrate that the fluid has an exceedingly-potent gluten detoxification capacity, which can be attributed to the combined action of a novel prolyl endoprotease and a non-canonical aspartic protease.

## Results

**Figure 1:**
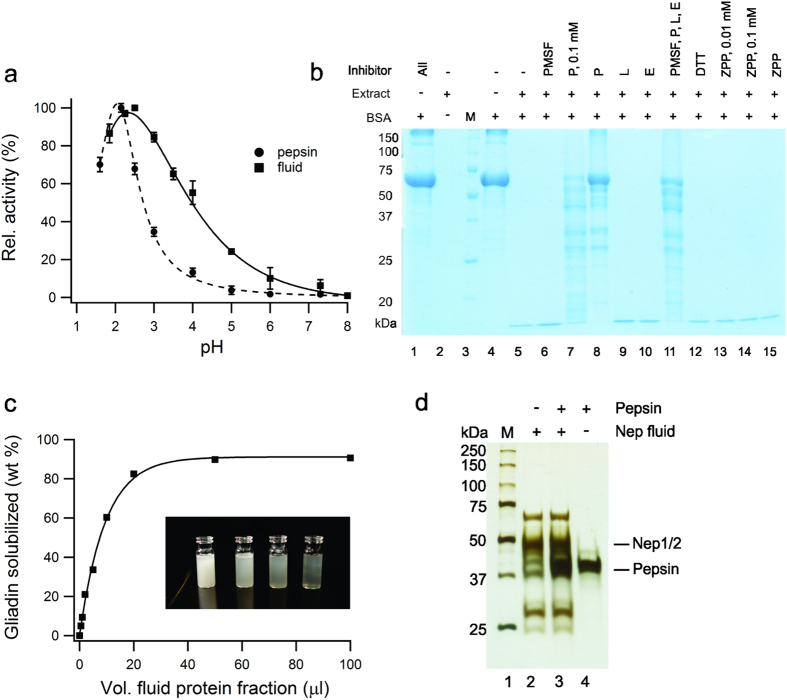
Proteolysis can be achieved at low pH using a concentrated protein extract from the fluid of *Nepenthes* carnivorous plants. (**a**) Relative digestion activity profile of concentrated fluid as a function of pH, compared with gastric pepsin (substrate: hemoglobin). (mean +/− std. dev. for n = 3). (**b**) Effect of proteolytic inhibitors on digestion of bovine serum albumin (BSA) measured by SDS PAGE. Inhibitors: PMSF (phenylmethylsulfonyl fluoride), P (pepstatin), L (leupeptin), E (EDTA and EGTA), DTT (dithiothreitol), ZPP (Z-Pro-Prolinal). All inhibitor concentrations at 1 mM except where noted. Extract: fluid protein extract, M: lane markers. (**c**) Gravimetric analysis of crude gliadin slurry digested with aliquots of extract. Inset: temporal profiling of slurry (left to right: 0, 30, 60, 90 min) using the protein-enriched fluid extract. (**d**) Silver-stain SDS PAGE of protein-enriched fluid showing the limited complexity of the fraction, and its high proteolytic stability in the presence of pepsin.

Based on a preliminary examination of the digestive properties of the *Nepenthes* secretions^31^, and the fact that the genus is adapted to nutrient adsorption from heterogeneous mixtures^28^, we speculated that the secretions may be effective at digesting poorly soluble wheat gluten proteins. However, in nature, the secretions require weeks for complete processing of prey. Correspondingly, we saw minimal digestion of protein standards in fluid harvested from a variety of *Nepenthes* species in a laboratory setting, at least under short digestion time-periods (<1 hr). Suspecting that the reason was a low enzyme concentration, we produced 100 *Nepenthes x ventrata* plants (~1000 individual pitchers), and stimulated the pitchers repeatedly over a six month period. Approximately 5 L of fluid was collected in this exercise. We recovered and concentrated the protein fraction in low pH buffer (pH 2.5). This concentrated fraction possessed high proteolytic activity against soluble protein standards (Fig S1) and remained stable for weeks at 4 °C, and under freeze/ thaw cycles. Using a classical hemoglobin assay for digestion^38^, we found that the concentrate exhibited maximum activity at pH 2.5 and retained activity up to pH 5 (Fig. 1a), encompassing a typical pH range for the human stomach. The pH profile exhibited some similarity to pepsin, but digestion could not be solely attributed to the aspartic protease components known to be present in the fluid (Figs 1b and S1). Pepstatin, an aspartic protease inhibitor, could only partially suppress enzyme activity. However, no other inhibitors appeared to have an effect.

**Figure 2:**
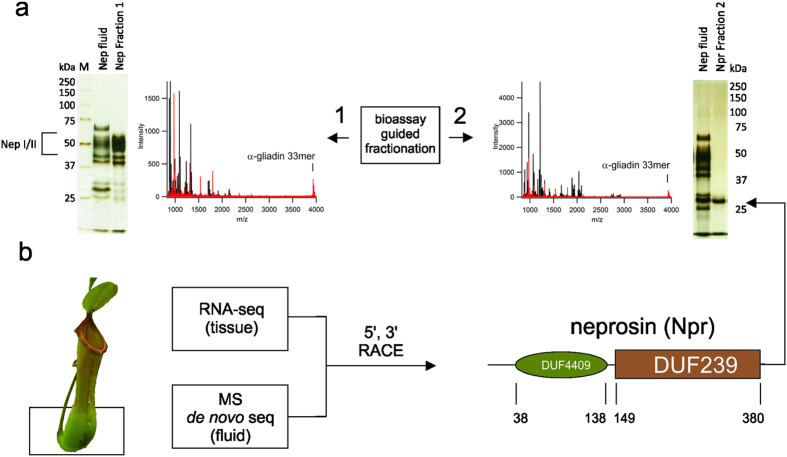
*Nepenthes* digestive fluid contains a novel protease consisting of two domains of unknown function. (**a**) Isolation of the two fractions from the activated fluid extract with proteolytic properties. Left: Silver-stain SDS-PAGE of fraction 1 enriched in nepenthesin I and nepenthesin II, and MALDI spectrum of 33mer peptide processed with fraction 1. Right: Silver-stain SDS-PAGE of fraction 2 containing an unknown enzyme, and MALDI spectrum of 33mer peptides processed with fraction 2. MALDI of undigested 33mer in red, digested 33mer in black. (**b**) Sequence identification strategy identifies fraction 2 enzyme as a two-domain construct, assigned the name *neprosin* in this study.

We then prepared a slurry of crude wheat gliadin, and demonstrated that the protein fraction of the fluid could rapidly clarify the slurry at pH 2.5 (Fig. 1c). The results were sufficiently compelling to warrant a closer functional analysis of the protein extract, which contained a plant sub-proteome of limited complexity that remained stable in the presence of pepsin (Fig. 1d). The enriched protein fraction was separated using bioassay-guided column fractionation, and two proteolytic components were isolated (Fig. 2). Each fraction was able to digest the peptide LQLQPFPQPQLPYPQPQLPYPQPQLPYPQPQPF, a 33mer sequence derived from α-gliadin that spans six overlapping T-cell epitopes and stimulates a potent T-cell response in celiac patients^4^,^39^. This peptide sequence is highly resistant to digestion by gastric pepsin, even with prolonged periods, which we confirmed using a control digestion.

The digestion of 33mer by fraction 1 appears due to the action of the aspartic protease in the fluid. We have shown previously that nepenthesins I and II have non-canonical cleavage properties for aspartic proteases^40^,^41^. Using proteomics techniques and searching available databases, we were able to confirm the presence of nepenthesin I in the protein extract (Tables S1 and S2). We suspected that conventional bottom-up proteomics methods, based on tryptic digestion, may not be ideal for the fluid. A sequence analysis of nepenthesin I showed only one tryptic cleavage site, suggesting that other components might similarly be difficult to identify with this approach. We replaced trypsin in the standard proteomic workflow with active fluid protease, and confirmed the presence of nepenthesin II in the fluid as well (Table S3). Purified fraction 1 analyzed in the same fashion identified only nepenthesin II.

We found no hits when fraction 2 was analyzed using a trypsin or active fluid digestion strategy. Peptide masses detected after processing the 33mer with fraction 2 (Fig. 2a) corresponded to proline-terminating fragments. Our earlier studies showed that nepenthesin I and II reconstituted all cleavage sites detected in the fluid, with the exception of Pro-X^40^,^42^. Given that a sequenced genome for *Nepenthes* is unavailable, we implemented a different strategy to identify what appears to be a proline-active enzyme. Gel analysis of the fraction suggested a simple composition (Fig. 2a), so we combined whole-transcriptome shotgun DNA sequencing with *de novo* sequencing of peptides that were generated by the non-specific digestion of the protein fraction. We assembled a 4 kDa segment from overlapping peptides and searched the transcriptome data to reveal a contig that, upon expansion with 5′ and 3′ RACE, coded for a protein represented by two domains of unknown function: Pfam entries DUF239 and DUF4409 (Fig. 2b). Mass analysis supports a mature enzyme consisting primarily of DUF239 (Fig. S2). A functional prediction for DUF239 in Pfam suggests C-terminal peptidase activity, but this has never been demonstrated. The level of sequence identity shared with other members possessing a DUF239 domain, mostly found in plants, is only modest (Fig. S3). Interestingly, this enzyme is both structurally and functionally distinct from known proline-cleaving enzymes (Fig. S4). We therefore conclude that DUF239 represents a previously unknown class of proline-directed protease. We explored its activity in greater detail using large protein standards, and generated proteolytic maps that clearly indicate an endoprotease with dominant Pro-X cleavage specificity (Fig. S5). At 29 kDa, the enzyme is considerably smaller than any known prolyl endoproteases, and does not appear to have the substrate length restrictions observed with prolyl endopeptidases^43^. We have named this newly-discovered proteolytic enzyme *neprosin* (Npr1).

Before testing the extract for gluten detoxification potential, and given the importance of dosage to the therapeutic concept, we measured the concentration of the active proteolytic components. We used a method involving AQUA-peptides, in which stable isotope-labeled peptides representing the active enzymes were added as internal standards^44^. The AQUA method requires identifiable tryptic peptides and complete digestion. Only nepenthesin I and neprosin could be monitored in this fashion, as mature nepenthesin II has no K or R residues. Complete digestion was achieved using an aggressive denaturing and digestion protocol. We determined nepenthesin I to be present at 450 +/− 50 nM (n = 3) and neprosin at 250 +/− 40 nM (n = 3) in the concentrated fluid fraction. Based on the label-free proteomics data that we collected from the whole fluid sub-proteome, using the nonspecific digestion protocol, we estimate that the concentration of nepenthesin II is approximately equivalent to nepenthesin I (see methods). For the purposes of dose evaluation in the gluten detoxification experiments, we approximated the total enzyme concentration in the concentrate at 1.15 μM, consisting of 900 nM nepenthesin I/II and 250 nM neprosin.

**Figure 3:**
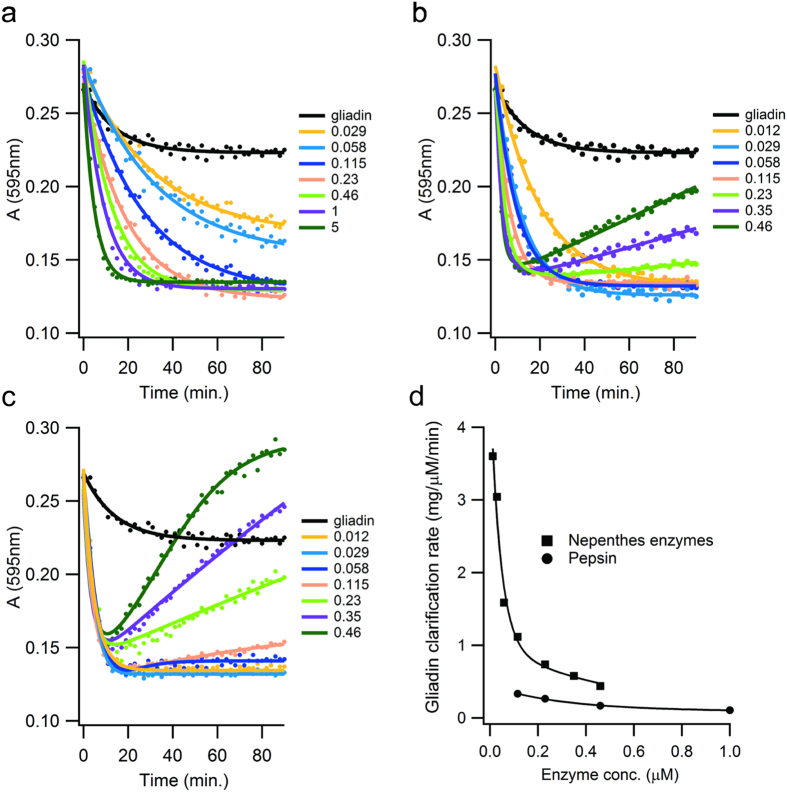
*Nepenthes* fluid proteases accelerate the clarification rate of gliadin slurries. (**a**–**c**) Time-course digestions of 10 mg/ml gliadin (pH 2.5 at 37 °C) with the indicated micromolar concentrations of (**a**) pepsin, (**b**) *Nepenthes* fluid proteases, or (**c**) *Nepenthes* fluid proteases supplemented with 5 μM pepsin. Turbidity of the digested gliadin was monitored as absorbance at 595 nm (A_595_). Black lines in (**a**–**c**) represent the turbidity of the gliadin slurry in the absence of protease. Measurement precision was <3% RSD (n = 3). (**d**) Slurry clarification rates, measured at 90% of the maximal effect, for digestions using *Nepenthes* fluid enzymes alone, and pepsin alone. Data taken from (**a**,**b**). Values reported as milligrams of gliadin per micromolar enzyme concentration per minute digestion time.

Next, we monitored the digestion characteristics of crude gliadin over a range of *Nepenthes* enzyme concentration. Digestion products were compared to pepsin-generated peptides as a control, using a number of methods. Digestion was performed at a pH of 2.5 in order to simulate gastric conditions alone. We did not implement a second-stage of digestion at neutral pH using intestinal proteases (*i.e*. trypsin, chymotrypsin). First, we monitored clarification rates using a 10 g/L slurry of a crude gliadin extract, by optical density measurements (Fig. 3a–c). Pepsin was applied up to the higher end of the human gastric concentration range (~5 μM)^45^, and fluid proteases to approximately 1/10^th^ of this amount. Pepsin alone achieved a maximum clarification rate of 0.3 mg/μM/min (Fig. 3d). We note some residual opacity that may be due to water-insoluble high molecular-weight glutenins or typical residues from the extraction process, such as lipids. The fluid proteases alone increased the clarification rate over 10-fold (Fig. 3d). Interestingly, at the higher levels of fluid proteases used in this test, opacity increased with digestion time, and co-digestion with pepsin amplified the effect. This finding is consistent with the emulsifying properties of gluten hydrolysates^46^. The stabilization of lipids in the crude fraction by an emulsion would increase scattering and would rise as digestion progresses. The effect prevented us from measuring the clarification rate using the combination of pepsin and fluid proteases. Nevertheless, the clarification rate of the fluid extract is high, and the fluid proteases appear to digest gliadin synergistically with pepsin.

**Figure 4:**
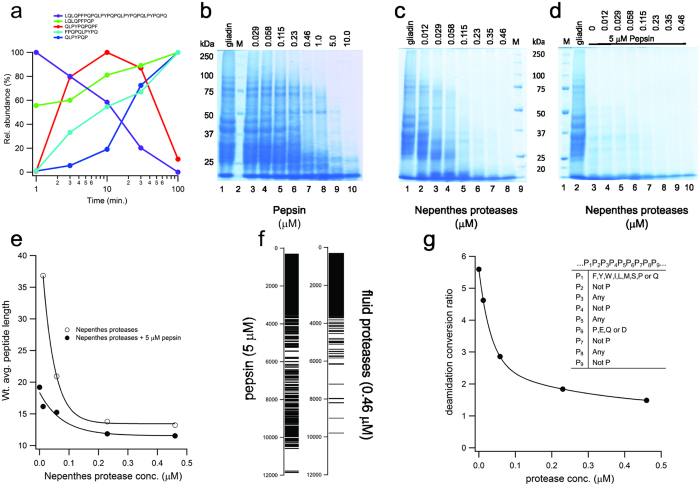
*Nepenthes* fluid proteases degrade crude gliadin and reduce antigenic load. (**a**) Digestion profile of α-gliadin peptide 33mer at 20:1 (substrate:enzyme), using fluid proteases under dilute conditions. Normalized relative abundance measured from LC-MS ion chromatograms, using caffeine as internal standard. (**b**–**d**) SDS-PAGE of 10 mg/ml gliadin digested with the indicated concentration of (**b**) pepsin, (**c**) fluid proteases or (**d**) pepsin in combination with fluid proteases. Digestions at pH 2.5 and 37 °C, for 90 min. All concentrations in μM. M: Molecular weight markers, and gliadin: total crude gliadin in the absence of protease. (**e**) Weighted average peptide length as a function of fluid protease concentration, with or without pepsin, using ion chromatogram intensities for weighting. Peptide data from LC-MS/MS runs, with intensities obtained using Protein Deconvolution 1.0. (**f**) Barcode representation of deconvoluted spectra from (**e**), at the noted enzyme concentrations, showing molecular weight distribution with simple binary model of intensities, (white: <0.2%, black: >0.2%). (**g**) TG2-induced conversion of gliadin digestion products to deamidated counterparts. Fractional deamidation quantified for all peptides from ion chromatogram intensities; data presented as a ratio, where the antigenic peptide deamidation is normalized to the non-antigenic peptide deamidation at the indicated protease concentration. Antigenic regions defined using the DQ2 criteria^11^,^39^, inset table.

These results suggest an effective digestion process, but they do not convey information regarding the completeness of the digestion. Using the pepsin-resistant 33mer, we monitored the evolution of peptide products under dilute conditions (Fig. 4a). At 10 min, approximately 50% of 33mer was digested, and full digestion was achieved at 100 min. To extend these findings, we carried out an extensive mapping of slurry digestion products using proteomics techniques. First, SDS-PAGE showed extensive digestion of total protein using fluid proteases, which could not be achieved with pepsin alone (Fig. 4b–d). Next, data-dependent LC-MS/MS data were collected on the digest products to globally characterize peptide size and sequence. Using a label-free method that can quantify all peptide signals from LC-MS data regardless of identity, we observed that a high concentration of pepsin (5 μM) hydrolyzes gliadin to a moderate level as expected (Fig. 4e), yielding an average peptide length of 19.2 residues. The fluid extract saturates at an average length of 13.2 residues using much lower doses (0.23 μM). With pepsin co-digestion the average length reduces further to 11.5 residues. The fluid proteases also generated a narrower distribution of product length (Fig. 4f). High concentrations of pepsin produce a digest with 25 wt% of the detected product having a molecular weight greater than 4000 Da, confirming the proteolytically-resistant properties of gluten. The fluid proteases reduce this fraction to less than 2 wt%. An alternative quantitation method that used only the peptide sub-set identified by the MS/MS data supports these findings (see supplementary data – MS/MS quantitation).

Inspecting the peptide sequences, we identified large numbers of Pro-X cleavage sites. To test if neprosin alone was responsible for generating the significant increase in digestion efficiency, we digested the crude gliadin slurry using purified neprosin. We observed a bimodal distribution of products, consisting of both low and high molecular weight fractions (Fig S6). The weighted average peptide length for the low molecular weight fraction was 12.5, accounting for 10% of the total signal, but doubling the concentration did not diminish the bimodality, nor significantly improve the depth of coverage. The high molecular weight fraction remained substantial, confirming that the aspartic proteases have a role in accelerating digestion. We reconstituted the natural aspartic protease to neprosin ratio, using recombinant nepenthesin II produced with previously described methods^40^ and the purified neprosin, and found that the gliadin digestion profile was equivalent to that of the fluid extract. It confirms that the proteolytic activity of the extract arises from the aspartic proteases and neprosin alone.

To gauge the potency of the *Nepenthes* enzymes against other enzymes, we first tested 33mer processing. Neither pepsin nor a bacterial prolyl endoprotease from *Myxococcus xanthus* (MX)^47^ were able to hydrolyse the peptide to any appreciable extent, in equivalent digestion conditions at their optimum pH (2 and 7 respectively). Next, we compared the *Nepenthes* enzymes with AN-PEP in a gliadin slurry digestion. We used the SDS-PAGE assay for an assessment of protein digestion and the turbidometric assay for the assessment of slurry clarification (Fig. S7). We required approximately 25 times more AN-PEP to achieve a comparable reduction in intact protein levels and over 1000 times more AN-PEP to achieve a comparable level of slurry clarification. The different ratios reflect different digestion endpoints in the assays. The gel analysis profiles the generation of larger digestion products, whereas the turbidometric assay profiles all digestion products.

We next investigated if the enhanced digestion profiles observed with the *Nepenthes* enzymes would impact deamidation in antigenic regions. The recognition of immunodominant peptides by T cells is amplified when these peptides are deamidated in their core binding region by tissue transglutaminase 2 (TG2), particularly at position P4 or P6 in HLA DQ2 associated celiac disease^48^. Deamidation is dependent on both peptide sequence and length, and the deamidation levels for key antigenic regions are significantly higher than elsewhere in sequence^48^. We analyzed the crude gliadin digests for the conversion of non-deamidated peptides to deamidated forms, and sorted all the peptides using a previously published algorithm for the identification of DQ2 binding motifs^11^,^39^. As shown in Fig. 4g (and Table S5), pepsin digestion generated over 5.5 times higher deamidation in antigenic regions, when compared to non-antigenic regions. By applying increasing concentrations of the fluid proteases, the conversion levels in the antigenic regions approached those of the non-antigenic sequences, especially when considering the relative Q content for each category of peptide (a conversion ratio of 1.3 is expected based on the higher Q content in antigenic peptides alone, at high enzyme concentration).

**Figure 5:**
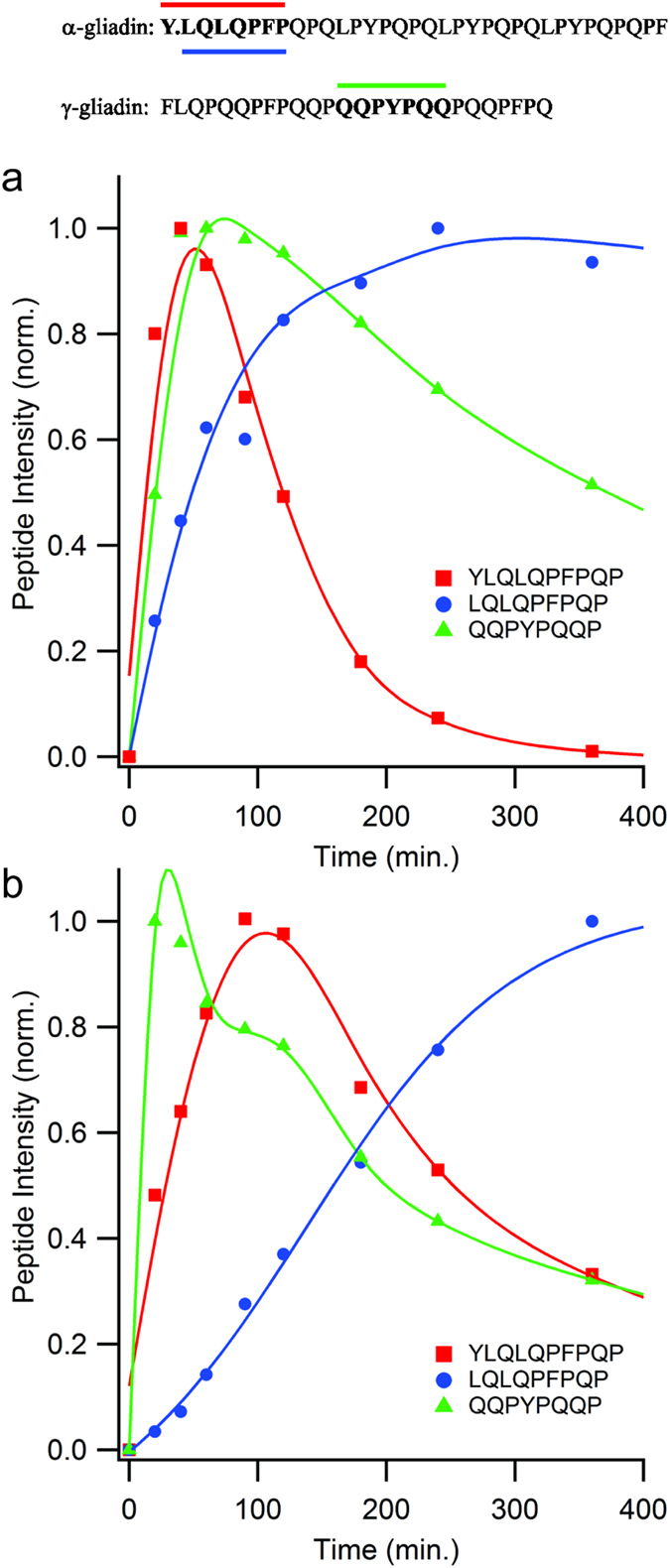
Antigen fragmentation remains efficient in the presence of excess non-gluten protein substrate loads. Temporal digestion profiles of three peptides (top) spanning the antigenic regions of α and γ-gliadin. (**a**) 10 mg/ml crude gliadin slurry supplemented with 1.15 μM fluid proteases (4:1 nepenthesins to neprosin) and 5 μM pepsin, at t = 0. (**b**) 10 mg/ml crude gliadin slurry and 90 mg/ml bovine serum albumin supplemented with 1.15 μM fluid proteases (4:1 nepenthesins to neprosin) and 5 μM pepsin, at t = 0. Data color coded with the peptide designations at the top, representing average determinations (n = 3, relative standard deviations <2%), standardized against stable-isotope labeled versions of the peptide, and reported as values normalized to the maximum intensities.

Based on these analyses, we calculate that an extensive digestion of crude gliadin slurry can be achieved using a substrate to fluid protease ratio of 1265:1, where the enzyme consists of a 4:1 blend of nepenthesin to neprosin. To test the influence of added non-gluten protein on the effectiveness of gluten detoxification, we performed a digestion experiment where gliadin consisted of only 10 wt% of the total, by adding 90 g/L serum albumin to the 10 g/L crude gliadin slurry. Fluid protease was maintained at 1.15 μM and pepsin at 5 μM. The excess of albumin prevented us from using data-dependent proteomics methods, so we monitored select peptides from the antigenic regions of α and γ-gliadin, using a targeted proteomics methods and AQUA peptides as internal standards (see methods). Applying the method against an albumin-free slurry digest confirmed the fragmentation of the antigenic regions (Fig. 5a), as described above, and shows that antigen processing is extensive by 60 min. The digest containing the large excess of albumin extended the α-gliadin antigen fragmentation timeframe only two-threefold, and it had no negative effect on the fragmentation of the γ-gliadin antigen.

**Figure 6:**
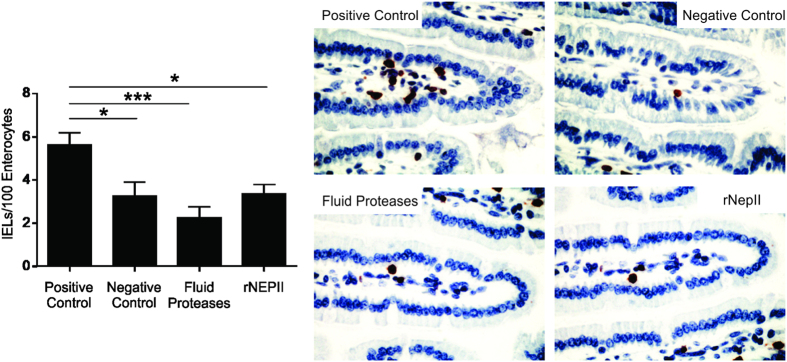
*Nepenthes* fluid protease treatment of gliadin prevents gliadin-induced inflammation in the NOD/DQ8 transgenic mouse model of gluten-sensitivity. Intraepithelial lymphocyte (IEL) counts quantitated by immunostaining of proximal small intestine villi tips, in sensitized mice challenged with pepsin-treated gliadin (positive control), vehicle alone (negative control, gliadin-free diet), fluid protease-treated gliadin (1:264 ratio) and recombinant nepenthesin-II treated gliadin (1:100 ratio). Representative immunostained sections of intestinal tissues shown to the right at 40x magnification. Statistical significance (n = 8 for each state, *p < 0.05, ***p < 0.001).

Finally, we tested the efficacy and tolerability of the fluid protease extract in mice transgenic for the HLA-DQ8 molecule in a Non-Obese Diabetic (NOD) background that confers susceptibility to autoimmunity (NOD/DQ8). This mouse model exhibits gluten-induced pathology in a DQ8-dependent manner^49^,^50^,^51^. Mice were sensitized to gliadin (see methods), and subsequently underwent oral gavage 3 times a week, for 2 weeks, using either pepsin-treated gliadin (P-gliadin, positive control), gliadin-free vehicle (negative control), gliadin treated with the enriched fluid protease fraction, or recombinant nepenthesin-II (Fig. S8). As expected, mice receiving P-gliadin developed increased intraepithelial lymphocyte (IEL) counts in the villi tips, as quantified by CD3^+^ cells, in comparison to mice receiving vehicle alone. In contrast, mice receiving the gliadin treated with the enriched fluid protease fraction or recombinant nepenthesin-II did not develop increased IELs (Fig. 6). Surprisingly, these results show that supplementation with a non-canonical aspartic protease alone can induce a measurable effect, although the presence of neprosin likely improves efficacy.

## Discussion

Species of the genus *Nepenthes* have adapted to growth in nitrogen-deficient conditions by attracting and trapping nitrogen-rich prey within the lower reaches of the pitcher leaf, a region that contains secretions capable of digesting invertebrates, plant matter, and animal waste. The pitcher achieves digestion without the benefit of mastication, in a single stage equivalent of a whole mammalian digestive tract. Chitinases, phosphatases, ribonucleases and proteases are involved in breaking down prey^28^, but remarkably little is known about the specific enzymatic components driving this impressive feat of nutrient capture and uptake. The unusual amino acid composition of the protein components and the lack of available sequence databases have prevented a thorough proteomic characterization. Two aspartic proteases uncovered during initial investigations (nepenthesin I and II) have received the most study to date^52^, and have been presumed to define the proteolytic capacity of the fluid. We noticed that the fluid possessed a cleavage specificity profile most unusual for aspartic proteases alone^31^. We established that the nepenthesins are non-canonical in their cleavage specificity^40^,^42^, cleaving after hydrophobic residues as pepsin does, but also possessing an unusual tryptic and chymotryptic character at low pH. We observed a robust C-terminal proline cleavage activity in the fluid as well, which was not reconstituted by the aspartic proteases alone^40^,^41^. These findings stimulated our interest in evaluating the secretions for use in gluten detoxification, and we set out to uncover the enzyme responsible for proline cleavage.

Our current study demonstrates that the proline cleavage characteristic that we observed in the fluid is separable from the action of the aspartic proteases, and together comprises a potent gluten digestion profile. Neprosin is the first characterized member of what appears to be a new class of prolyl endoprotease, defining a core function for a protein domain that is well represented in plants, but not functionally annotated until this study. The sequence identity shared with other members of DUF239 is only modest, suggesting that, like the aspartic proteases, this prolyl endoprotease may have certain properties outside the norm for the class. Further research into other members of DUF239 is warranted. Nevertheless, while neprosin is important for a robust digestion profile, the plant aspartic proteases contribute to an efficient process. The surprising reduction in intestinal inflammation that we observed when combining nepenthesin II alone with pepsin highlights the non-equivalent nature of these aspartic proteases. Pepsin is also more effective at gliadin digestion in the presence of fluid proteases (Fig. 4e), suggesting the possibility of synergy between host and plant proteases.

Our findings widen the possibilities for treating celiac disease through an enzyme supplementation strategy. Although the concept of enzymatic digestion has been presented^19^,^23^, and several companies have marketed digestion aids based on the concept, it has been shown conclusively that none of the available options are likely to be effective, either because of dose restrictions or inappropriate cleavage specificity^18^. AN-PEP, a protease in the general prolyl endoprotease functional class, appears to be the most effective agent to date, but it requires administration in amounts that are nearly equivalent to the gluten proteins consumed^18^,^53^. In our study, we demonstrate that the range of effectiveness for the concept can be extended considerably. Very low nepenthes enzyme levels strongly enhance the solubilization rate for gliadin slurries, which seems to us an important requirement for an effective supplementation strategy. A soluble form is necessary for effective digestion in the stomach, regardless of the choice of enzyme. We suggest that supplementation with a non-specific aspartic protease in the nepenthesin class is a key element of any concept requiring gastric digestion, and that a prolyl endoprotease (as a stand-alone supplement) may be insufficient. Efficient protein breakdown will be required before a further reduction of gluten proteins can be effective, which may be the role of nepenthesin. Our finding that nepenthesin supplementation alone leads to reduced inflammation, at higher doses than the fluid proteases but still very low relative to current alternatives, is particularly interesting. The non-canonical nature of this aspartic protease appears to be a contributor to efficacy, indicating that cleavage sites other than C-terminal proline can be targeted. As the efficacy threshold for an effective supplement is high, given the variable size and complex nature of meals, both enzymes will likely be required in combination.

When blended at the natural levels that we measured, a high antigen breakdown capacity is preserved, even when the ratio is over 12,000:1 (total protein to enzyme). This represents less than 5 milligrams of enzyme for a 50 gram total daily protein load. Clearly, clinical studies are required to ascertain if this very low level of supplementation will be effective, but the extensive biochemical analysis we conducted in this study suggests that it is possible. With alternative blends and modest increases in dosage, it may be possible to support an effective adjuvant therapy or even an alternative to a gluten-free diet in the treatment of celiac disease.

## Materials and Methods

### Horticulture

*N. ventrata* (100 plantings in 8” pots) were grown in a dedicated greenhouse (Urban Bog, Langley, BC, Canada). The plants were potted with wood bark, perlite, peat moss and humus mix (40, 35, 10, 5% respectively) and grown under natural lighting, with controlled humidity and temperature. Irrigation was applied at the soil level, to avoid addition of water to the pitchers. The pitchers were fed with frozen *Drosophila spp*., 1 or 2 in every pitcher (~1000 pitchers), although we note that insects were also harvested by the plants from the environment (e.g. wasps). Fluid was harvested the following week by pipette, and the cycle repeated until 5 litres of fluid was collected. Crude pitcher fluid was clarified using a 0.22 μm filter and the protein fraction concentrated 10X using an Amicon 10 kDa cut-off spin filter (Millipore), and washed 3X with 100 mM glycine-HCl (pH 2.5, active conditions) to remove any peptides resulting from self-digestion or residual prey digestion.

### Standard activity assays

For pH profiling, proteolytic activity was measured using a modified version of the hemoglobin activity assay^38^. The assay consisted of 4 μL concentrated *Nepenthes* fluid mixed with 1.25 mg equine hemoglobin (Sigma Aldrich) to a final volume of 100 μL in an appropriate buffer. Protein was digested for 30 minutes at 37 °C, 200 rpm, and quenched with 10% TCA. The precipitate was removed by centrifugation (14000 *g*, 10 minutes) and the supernatant used to measure the absorbance of soluble peptides at 280 nm (GE Nanovue plus spectrophotometer). All data points are the means of three technical and three biological replicates. For enzyme inhibition testing, bovine serum albumin (1 mg/ml) was digested with 0.12 μM *Nepenthes* enzymes for 15 min at 37 °C, quenched by brief boiling and then analyzed by 10% SDS-PAGE. The enzymes were pretreated with controls and inhibitors at 4 °C for 2 days prior to digestion experiments. PMSF, pepstatin, leupeptin, EDTA, EGTA, DTT from Sigma-Aldrich, and ZPP from Bachem.

### Bioassay-guided fractionation

Fluid protein concentrate was exchanged into 50 mM glycine and subjected to column chromatofocusing with fractionation. Fractions were analyzed for activity using MALDI-TOF (Sciex 5800 TOF/TOF, α-cyano-4-hydroxycinnamic acid as matrix). To test for activity, protein substrates were incubated with aliquots of column fractions at room temperature for 20 minutes and the digests analyzed by MALDI-TOF. Fractions enriched in proline cleavage were manually purified by reversed phase chromatography on Protein MacroTraps (Optimize Technologies). Enzyme was eluted and confirmed active against protein substrates. Activity was retested against 33mer from α-gliadin using MALDI-TOF. Fractions were also analyzed for protein content using MALDI-TOF (sinapinic acid as matrix), highlighting a single peak at 29 kDa. Fractions enriched in non-specific cleavage properties were further purified using gel filtration and retested for purity and for activity against 33mer.

### Plant protein identification and quantitation

Fluid concentrate and column fractions were analyzed using gel-free and gel-based proteomics methods. For gel free methods, proteins were reduced with DTT and alkylated with iodoacetamide using standard methods. Denatured samples were digested overnight with trypsin or for 1 hour with concentrated *Nepenthes* fluid. The digests solutions were lyophilized and resuspended in 1% formic acid (FA) prior to injection in the mass spectrometer for data-dependent LC-MS/MS analyses. For protein deglycosylation, sample was treated with PNGase F (New England Biolabs) following the manufacturer’s method, prior to tryptic digestion. For gel-based proteomics analysis of fluid fractions, protein was separated by SDS-PAGE, and bands selected for in-gel tryptic digestion and data-dependent LC-MS/MS of the products. After washing, reduction and alkylation, the gel pieces were incubated overnight with trypsin, then extracted for mass analysis.

Protein digests were separated using an LC system (Easy-nLC 1000, Thermo Scientific) operating in a nanoflow configuration. Peptides were selected in a top-10 data-dependent experiment for collision-induced dissociation (CID) on a Thermo Orbitrap Velos ETD mass spectrometer in a high/low configuration (MS: Orbitrap at 60,000 resolution and MS/MS: ion trap). The data were searched using Mascot v2.3 (Matrix Sciences) against the NCBI Viriplantae (green plants), *Drosophila* and Bacteria/eubacterial databases, using conventional settings for tryptic digestion. For fluid-based digestions, the searches were configured using “no enzyme” specificity and other settings remained the same. For the identification of neprosin, peptides generated using the fluid extract were sequenced *de novo*, with the assistance of PEAKS software v7.1, producing a 4 kDa sequence with an accuracy >90%, and searched against the *Nepenthes* transcriptome (see below).

For enzyme quantitation, a variation of the FASP protocol^54^ was applied to digest the fluid concentrate, and combined with the AQUA peptide quantitation method^55^. Briefly, heavy-labeled peptides for nepenthesin I (GPLSLPSQLDVTK) and neprosin (ASYVR) were synthesized (Sigma-Aldrich). The fluid concentrate was denatured in 8 M urea at neutral pH and under reducing conditions. Protein was alkylated with iodoacetamide, and then digested with trypsin. AQUA peptides were added, and the samples were then purified for mass analysis. Digests were analyzed by reverse-phase LC-MS on an Orbitrap Velos ETD. The relative intensities of the light and heavy forms of the tryptic peptides measured in Xcalibur software and used to determine protein concentration. Progressively-longer digestion times and higher enzyme-to-substrate ratios were applied until protein concentrations reached a plateau. To estimate nepenthesin II levels required label-free methods (emPAI^56^ and T3PQ^57^) applied to the nonspecific digest of the fluid protease fraction.

### Transcriptomics and 5′ and 3′ RACE

*N. ampullaria* were lab-grown in a small terrarium, in a 15–9 h light-dark photoperiod. On pitcher maturity, the plants were fed with 1 or 2 *Drosophila spp*. per pitcher 24 hours before RNA extraction. For RNA extraction, the digestive fluid was removed and the pitchers washed with deionized water to remove partially digested material and other debris. The bottom one-third of the pitcher containing the secretory cells was excised, and frozen in liquid nitrogen. The bottom section of the pitcher was ground to a fine powder under liquid nitrogen and the total RNA extracted using a modified CTAB protocol^58^.

SOLiD sequencing of the *N. ampullaria* transcriptome was performed by the University of Calgary Genomics Facility (Calgary, AB). Poly(A) RNA was enriched from 10 μg of total RNA using a Micro Purification Kit (Dynabeads mRNA DIRECT, Life Technologies), following the manufacturer’s protocol. Whole transcriptome RNA libraries were prepared from 25 ng of polyA-captured RNA using the SOLID Total RNA-Seq kit (Life Technologies). After fragmentation by chemical hydrolysis, adapter ligation and reverse transcription using SOLiD primers, the cDNA was purified and size-selected (Agencourt AMPure XP beads) then PCR amplified. The cDNA library was sequenced on an ABI SOLID 5500 sequencer (Life Technologies) using paired-end 75 + 35 bp runs. For analysis, raw sequencing data was converted to csFastq using SOLiD’s XSQ_Tools and local Perl scripts. Cleaning filters were applied to data files, based on FastQC quality control statistics, and SOLiD sequencing adaptors detected with Cutadapt. Reads were clipped using TrimmomaticPE at a threshold of Phred 20, with a sliding window of 4, and returning clipped sequences at a minimum length of 25 bp. Only those reads were kept where both forward and reverse reads were retained. These cleaned read pairs were converted to pseudo-base space for assembly. *De novo* transcript assembly at various k-mers was performed with Velvet (version 1.2.10) and Oasis (version 0.2.8), compiled for colorspace. A final k-mer of 39 was used to assemble the combined forward read set of all sequence lanes in a single end assembly. Searching for the sequence tags generated by *de novo* MS/MS required the transformation of the assemblies into basespace. This was performed using utilities in SOLiD’s denovo2 pipeline package and local perl scripts. tBlastn was used to identify a partial contig of 628 bp that best matched the query. To determine the sequence upstream and downstream of the hit, we synthesized primers GATTACGCCAAGCTTCATTCCCGTTGGGATCTACGCATTG (LSO2R) and ACGACAACTCAGATGGGAAGCGG (LSO7F) for 5′ and 3′ RACE respectively (synthesized by the University of Calgary Core DNA Services). 5′ and 3′ RACE were performed using the SMARTer RACE 5′/3′ kit (Clonetech, Mountain View, CA) following the manufacturer’s protocol. Briefly, first strand cDNA was reverse synthesized using the manufacturer’s modified oligo (dT) and/or SMARTer II A oligonucleotide primers. 5′ and 3′ RACE PCR were amplified with Phusion high-fidelity DNA polymerase (New England BioLabs) using the SMARTer universal primer paired with the specific primers (LSO2R or LSO7R). The PCR involved 30 cycles of denaturing at 98 °C for 30 seconds, annealing at 62 °C for 30 seconds and extension at 72 °C for 90 seconds. The 1 kb 5′ RACE and 500 bp 3′ RACE PCR products were gel-purified, and sequenced by the University of Calgary Core DNA Services. We repeated 5′ and 3′ RACE on cDNA from *N. ventrata* and *N. rafflesiana* and found Npr1 sequences that were 97% identical.

### Gliadin slurry digestions

Crude gliadin (Sigma-Aldrich cat. # G3375) was ground to a powder. A stock gliadin slurry of 20–50 mg/ml was prepared in acidic solution (100 mM glycine HCl, pH 2.5) and briefly sonicated to further break up large particulates and promote suspension. Digestions of 10 mg/ mL gliadin slurries were initiated by addition of enzyme(s) and held at 37 °C with gentle rotation (buffered in 100 mM glycine-HCl, pH 2.5, except where noted). Digest progress was monitored by gravimetric analysis, optical transmission, SDS-PAGE and mass spectrometry. For gravimetric analysis, digests were quenched by boiling and treated with TCA/chloroform, followed by centrifugation to recover undigested or poorly digested protein. For monitoring with optical transmission, digestion reactions were conducted in 96-well plate with ≥3 replicates of each reaction condition, and turbidity monitored at 595 nm at 37 °C every 2 minutes, for 90 minutes (SpectraMax plate reader, Molecular Devices). The plate was shaken at medium speed briefly between measurements. The digests were quenched after 90 min reaction by boiling for 10 minutes (confirmed to have no impact on digestion profile, *a posteriori*). An aliquot of the reaction mixture was analyzed on an 8% SDS-PAGE gel. The remaining amount was centrifuged and the supernatant was analyzed by mass spectrometry (see below).

### Proteomic analysis of gliadin digests

Supernatant was analyzed by data-dependent LC-MS/MS, in two 1-hour reversed phase gradient runs, configured for top-10 ion selection using CID in a high/low configuration. In one run, ion selection was restricted to 2+ and higher charge states. In the other run, ion selection was applied to 1+ charge states only. Data from both runs were combined and searched against all UniprotKB/Swiss-Prot entries for gliadin and glutenin from *triticum aestivum* (25 proteins), using Mascot v2.3, configured for non-specific digestions and filtered for peptide hits with p < 0.05. For estimation of peptide size distribution, all LC-MS spectra were combined and deconvoluted in Protein Deconvolution v1.0, with appropriate settings. For label-free quantitative analysis using the subset of the digest identified in the database search, the hit list was combined with the raw data in Mass Spec Studio and used to generate a set of peptide ion chromatograms, integrated over all isotopes to determine a weight-average intensity for each peptide sequence identified.

To determine the impact of added protein on gliadin digestion efficiency, bovine serum albumin (BSA, 90 mg/ mL) was applied in excess over gliadin (10 mg/ mL) and digested using 0.46 μM fluid protease and 5 μM pepsin. Samples were digested at 37 °C, pH 2.5 and an aliquot removed at multiple timepoints for analysis. Samples were diluted and quenched by boiling. As a control, a similar course of reactions was performed in the absence of BSA. Prior to mass analysis of digests, fixed amounts of two AQUA peptides (YLQLQPFPQP and LQLQPFPQP) representing an antigenic region of α-gliadin and 1 AQUA peptide representing an antigenic region of γ-gliadin (QQPYPQQP) were added (heavy-labeled amino acids underlined). Multiple transitions for each peptide (light and heavy forms) were monitored using a scheduled MRM method on a reversed-phase LC-MS system (Eksigent microLC on a Qtrap 6500). Data was collected in triplicate for each digest and timepoint. Chromatographic peak intensities for the respective quantifier transitions were measured, and standardized against the corresponding transitions for the corresponding heavy peptide.

### TG2 assay and global analysis

Supernatants from crude gliadin digests were treated with 0.1 mg/mL human transglutaminase-2 (R&D Systems, Cat. 4376-TG-050) in 100 mM Tris-HCl pH 7.5, 2 mM CaCl_2_ at 37 °C for 90 minutes, and quenched at 95 °C for 15 minutes, following a published protocol^48^. Treated digests were analyzed by data-dependent LC-MS/MS as described above, allowing for variable N and Q deamidation in database searches. The chromatographic intensities of all positively identified deamidated and nondeamidated peptides were determined using Mass Spec Studio^59^ and ratios expressing the relative degree of deamidation determined on a per-peptide basis.

### Animal studies

Original breeding pairs of SPF NOD AB°DQ8 (NOD/DQ8) mice were kindly provided by Drs. Chella David and Joseph Murray of the Mayo Clinic. Female and male SPF NOD AB°DQ8 (NOD/DQ8) mice, maintained on a gluten-free diet and bred in a conventional SPF facility at McMaster University, were sensitized with cholera toxin (CT, Sigma-Aldrich) and pepsin-gliadin digest (P-G, 500 μg) via oral gavage, to break oral tolerance to gliadin. Mice were subsequently challenged over a three week period, three times a week (See Fig. S7) to induce intestinal immunopathology. During the challenge phase, sensitized mice received, via oral gavage, either P-G, vehicle, gliadin co-digested with pepsin and fluid proteases or gliadin co-digested with pepsin and recombinant nepenthesin II (n = 8/group), using 5 mg gliadin per gavage. Mice were sacrificed 24 hours after final challenge. Proximal small intestinal tissue was collected and fixed in 10% formalin and embedded in paraffin. Paraffin sections were stained via immunohistochemistry for CD3^+^ cells and intraepithelial lymphocytes quantified by counting CD3^+^ IELs per 20 enterocytes in 5 villi tips, as previously described^50^. All experiments conducted were approved by the McMaster University Animal Care committee, in accordance with the regulations of the Animals for Research Act of the Province of Ontario and the guidelines of the Canadian Council on Animal Care.

## Additional Information

**How to cite this article**: Rey, M. *et al*. Addressing proteolytic efficiency in enzymatic degradation therapy for celiac disease. *Sci. Rep*. **6**, 30980; doi: 10.1038/srep30980 (2016).

## Supplementary Material

Supplementary Information

## References

[b1] Abadie V., Sollid L. M., Barreiro L. B. & Jabri B. Integration of genetic and immunological insights into a model of celiac disease pathogenesis. Annu Rev Immunol 29, 493–525 (2011). 21219178 10.1146/annurev-immunol-040210-092915

[b2] Shewry P. R. Wheat. J Exp Bot 60, 1537–1553 (2009). 19386614 10.1093/jxb/erp058

[b3] Kasarda D. D. . Nucleic acid (cDNA) and amino acid sequences of alpha-type gliadins from wheat (Triticum aestivum). Proc Natl Acad Sci USA 81, 4712–4716 (1984). 6589619 10.1073/pnas.81.15.4712 PMC391560

[b4] Shan L. . Structural basis for gluten intolerance in celiac sprue. Science 297, 2275–2279 (2002). 12351792 10.1126/science.1074129

[b5] Bethune M. T. & Khosla C. Oral enzyme therapy for celiac sprue. Methods Enzymol 502, 241–271 (2012). 22208988 10.1016/B978-0-12-416039-2.00013-6 PMC3382113

[b6] Sollid L. M. & Lie B. A. Celiac disease genetics: current concepts and practical applications. Clin Gastroenterol Hepatol 3, 843–851 (2005). 16234020 10.1016/s1542-3565(05)00532-x

[b7] Tollefsen S. . HLA-DQ2 and -DQ8 signatures of gluten T cell epitopes in celiac disease. J Clin Invest 116, 2226–2236 (2006). 16878175 10.1172/JCI27620 PMC1518792

[b8] Sollid L. M. & Thorsby E. HLA susceptibility genes in celiac disease: genetic mapping and role in pathogenesis. Gastroenterology 105, 910–922 (1993). 8359659 10.1016/0016-5085(93)90912-v

[b9] Anderson R. P., Degano P., Godkin A. J., Jewell D. P. & Hill A. V. *In vivo* antigen challenge in celiac disease identifies a single transglutaminase-modified peptide as the dominant A-gliadin T-cell epitope. Nat Med 6, 337–342 (2000). 10700238 10.1038/73200

[b10] Molberg O. . Tissue transglutaminase selectively modifies gliadin peptides that are recognized by gut-derived T cells in celiac disease. Nat Med 4, 713–717 (1998). 9623982 10.1038/nm0698-713

[b11] Kim C. Y., Quarsten H., Bergseng E., Khosla C. & Sollid L. M. Structural basis for HLA-DQ2-mediated presentation of gluten epitopes in celiac disease. Proc Natl Acad Sci USA 101, 4175–4179 (2004). 15020763 10.1073/pnas.0306885101 PMC384714

[b12] Olsson C., Hornell A., Ivarsson A. & Sydner Y. M. The everyday life of adolescent coeliacs: issues of importance for compliance with the gluten-free diet. J Hum Nutr Diet 21, 359–367 (2008). 18754144 10.1111/j.1365-277x.2008.00867.x

[b13] See J. A., Kaukinen K., Makharia G. K., Gibson P. R. & Murray J. A. Practical insights into gluten-free diets. Nat Rev Gastroenterol Hepatol 12, 580–591 (2015). 26392070 10.1038/nrgastro.2015.156

[b14] Mahadev S., Gardner R., Lewis S. K., Lebwohl B. & Green P. H. Quality of Life in Screen-detected Celiac Disease Patients in the United States. J Clin Gastroenterol (2015). 10.1097/MCG.0000000000000433 26501877

[b15] Norsa L. . Gluten-free diet or alternative therapy: a survey on what parents of celiac children want. Int J Food Sci Nutr 66, 590–594 (2015). 26171630 10.3109/09637486.2015.1064872

[b16] Lahdeaho M. L., Lindfors K., Airaksinen L., Kaukinen K. & Maki M. Recent advances in the development of new treatments for celiac disease. Expert Opin Biol Ther 12, 1589–1600 (2012). 22928821 10.1517/14712598.2012.721766

[b17] McCarville J. L., Caminero A. & Verdu E. F. Pharmacological approaches in celiac disease. Curr Opin Pharmacol 25, 7–12 (2015). 26414923 10.1016/j.coph.2015.09.002

[b18] Janssen G. . Ineffective degradation of immunogenic gluten epitopes by currently available digestive enzyme supplements. PLos One 10, e0128065 (2015). 26030273 10.1371/journal.pone.0128065 PMC4452362

[b19] Stepniak D. . Highly efficient gluten degradation with a newly identified prolyl endoprotease: implications for celiac disease. Am J Physiol Gastrointest Liver Physiol 291, G621–629 (2006). 16690904 10.1152/ajpgi.00034.2006

[b20] Tack G. J. . Consumption of gluten with gluten-degrading enzyme by celiac patients: a pilot-study. World J Gastroenterol 19, 5837–5847 (2013). 24124328 10.3748/wjg.v19.i35.5837 PMC3793137

[b21] Salden B. N. . Randomised clinical study: Aspergillus niger-derived enzyme digests gluten in the stomach of healthy volunteers. Aliment Pharmacol Ther 42, 273–285 (2015). 26040627 10.1111/apt.13266 PMC5032996

[b22] Lahdeaho M. L. . Glutenase ALV003 attenuates gluten-induced mucosal injury in patients with celiac disease. Gastroenterology 146, 1649–1658 (2014). 24583059 10.1053/j.gastro.2014.02.031

[b23] Gass J., Bethune M. T., Siegel M., Spencer A. & Khosla C. Combination enzyme therapy for gastric digestion of dietary gluten in patients with celiac sprue. Gastroenterology 133, 472–480 (2007). 17681168 10.1053/j.gastro.2007.05.028

[b24] Tye-Din J. A. . The effects of ALV003 pre-digestion of gluten on immune response and symptoms in celiac disease *in vivo*. Clin Immunol 134, 289–295 (2010). 19942485 10.1016/j.clim.2009.11.001

[b25] Siegel M. . Safety, tolerability, and activity of ALV003: results from two phase 1 single, escalating-dose clinical trials. Dig Dis Sci 57, 440–450 (2012). 21948339 10.1007/s10620-011-1906-5

[b26] Wu G. . Proline and hydroxyproline metabolism: implications for animal and human nutrition. Amino Acids 40, 1053–1063 (2011). 20697752 10.1007/s00726-010-0715-z PMC3773366

[b27] Hooker J. D. The carnivorous habits of plants. Nature 10, 366–372 (1874).

[b28] Moran J. A. & Clarke C. M. The carnivorous syndrome in Nepenthes pitcher plants: current state of knowledge and potential future directions. Plant Signal Behav 5, 644–648 (2010). 21135573 10.4161/psb.5.6.11238 PMC3001552

[b29] Clarke C. M. . Tree shrew lavatories: a novel nitrogen sequestration strategy in a tropical pitcher plant. Biol Lett 5, 632–635 (2009). 19515656 10.1098/rsbl.2009.0311 PMC2781956

[b30] Buch F., Kaman W. E., Bikker F. J., Yilamujiang A. & Mithofer A. Nepenthesin protease activity indicates digestive fluid dynamics in carnivorous nepenthes plants. PLos One 10, e0118853 (2015). 25750992 10.1371/journal.pone.0118853 PMC4353617

[b31] Rey M. . Nepenthesin from monkey cups for hydrogen/deuterium exchange mass spectrometry. Mol Cell Proteomics 12, 464–472 (2013). 23197791 10.1074/mcp.M112.025221 PMC3567866

[b32] Zhang J. . Evaporation-induced transition from Nepenthes pitcher-inspired slippery surfaces to lotus leaf-inspired superoleophobic surfaces. Langmuir 30, 14292–14299 (2014). 25378097 10.1021/la503300k

[b33] Gorb E. V., Purtov J. & Gorb S. N. Adhesion force measurements on the two wax layers of the waxy zone in Nepenthes alata pitchers. Sci Rep 4, 5154 (2014). 24889352 10.1038/srep05154 PMC4042122

[b34] Ishisaki K., Honda Y., Taniguchi H., Hatano N. & Hamada T. Heterogonous expression and characterization of a plant class IV chitinase from the pitcher of the carnivorous plant Nepenthes alata. Glycobiology 22, 345–351 (2012). 21930651 10.1093/glycob/cwr142

[b35] Wong T. S. . Bioinspired self-repairing slippery surfaces with pressure-stable omniphobicity. Nature 477, 443–447 (2011). 21938066 10.1038/nature10447

[b36] Takeuchi Y. . *In situ* enzyme activity in the dissolved and particulate fraction of the fluid from four pitcher plant species of the genus Nepenthes. PLos One 6, e25144 (2011). 21949872 10.1371/journal.pone.0025144 PMC3174996

[b37] Mithofer A. Carnivorous pitcher plants: insights in an old topic. Phytochemistry 72, 1678–1682 (2011). 21185041 10.1016/j.phytochem.2010.11.024

[b38] Anson M. L. The Estimation of Pepsin, Trypsin, Papain, and Cathepsin with Hemoglobin. J. Gen. Physiol. 22, 79–89 (1938). 19873094 10.1085/jgp.22.1.79 PMC2213732

[b39] Shan L. . Identification and analysis of multivalent proteolytically resistant peptides from gluten: implications for celiac sprue. J Proteome Res 4, 1732–1741 (2005). 16212427 10.1021/pr050173t PMC1343496

[b40] Yang M. . Recombinant Nepenthesin II for Hydrogen/Deuterium Exchange Mass Spectrometry. Anal Chem 87, 6681–6687 (2015). 25993527 10.1021/acs.analchem.5b00831

[b41] Kadek A. . Aspartic protease nepenthesin-1 as a tool for digestion in hydrogen/deuterium exchange mass spectrometry. Anal Chem 86, 4287–4294 (2014). 24661217 10.1021/ac404076j

[b42] Kadek A. . Expression and characterization of plant aspartic protease nepenthesin-1 from Nepenthes gracilis. Protein Expr Purif 95, 121–128 (2014). 24365662 10.1016/j.pep.2013.12.005

[b43] Mika N., Zorn H. & Ruhl M. Prolyl-specific peptidases for applications in food protein hydrolysis. Appl Microbiol Biotechnol 99, 7837–7846 (2015). 26239067 10.1007/s00253-015-6838-0

[b44] Gerber S. A., Rush J., Stemman O., Kirschner M. W. & Gygi S. P. Absolute quantification of proteins and phosphoproteins from cell lysates by tandem MS. Proc Natl Acad Sci USA 100, 6940–6945 (2003). 12771378 10.1073/pnas.0832254100 PMC165809

[b45] Roberts N. B., Sheers R. & Taylor W. H. Secretion of total pepsin and pepsin 1 in healthy volunteers in response to pentagastrin and to insulin-induced hypoglycaemia. Scand J Gastroenterol 42, 555–561 (2007). 17454875 10.1080/00365520601010131

[b46] Joye I. J. & McClements D. J. Emulsifying and emulsion-stabilizing properties of gluten hydrolysates. J Agric Food Chem 62, 2623–2630 (2014). 24571632 10.1021/jf5001343

[b47] Fuhrmann G. & Leroux J. C. *In vivo* fluorescence imaging of exogenous enzyme activity in the gastrointestinal tract. Proc Natl Acad Sci USA 108, 9032–9037 (2011). 21576491 10.1073/pnas.1100285108 PMC3107327

[b48] Dorum S., Qiao S. W., Sollid L. M. & Fleckenstein B. A quantitative analysis of transglutaminase 2-mediated deamidation of gluten peptides: implications for the T-cell response in celiac disease. J Proteome Res 8, 1748–1755 (2009). 19239248 10.1021/pr800960n

[b49] Galipeau H. J. . Intestinal Microbiota Modulates Gluten-Induced Immunopathology in Humanized Mice. Am J Pathol 185, 2969–2982 (2015). 26456581 10.1016/j.ajpath.2015.07.018 PMC4630176

[b50] Galipeau H. J. . Sensitization to gliadin induces moderate enteropathy and insulitis in nonobese diabetic-DQ8 mice. J Immunol 187, 4338–4346 (2011). 21911598 10.4049/jimmunol.1100854 PMC3493154

[b51] Marietta E. . A new model for dermatitis herpetiformis that uses HLA-DQ8 transgenic NOD mice. J Clin Invest 114, 1090–1097 (2004). 15489956 10.1172/JCI21055 PMC522239

[b52] Tokes Z. A., Woon W. C. & Chambers S. M. Digestive enzymes secreted by the carnivorous plant Nepenthes macferlanei L. Planta 119, 39–46 (1974). 24442407 10.1007/BF00390820

[b53] Mitea C. . Efficient degradation of gluten by a prolyl endoprotease in a gastrointestinal model: implications for coeliac disease. Gut 57, 25–32 (2008). 17494108 10.1136/gut.2006.111609

[b54] Wisniewski J. R., Zougman A., Nagaraj N. & Mann M. Universal sample preparation method for proteome analysis. Nat Methods 6, 359–362 (2009). 19377485 10.1038/nmeth.1322

[b55] Kirkpatrick D. S., Gerber S. A. & Gygi S. P. The absolute quantification strategy: a general procedure for the quantification of proteins and post-translational modifications. Methods 35, 265–273 (2005). 15722223 10.1016/j.ymeth.2004.08.018

[b56] Ishihama Y. . Exponentially modified protein abundance index (emPAI) for estimation of absolute protein amount in proteomics by the number of sequenced peptides per protein. Mol Cell Proteomics 4, 1265–1272 (2005). 15958392 10.1074/mcp.M500061-MCP200

[b57] Grossmann J. . Implementation and evaluation of relative and absolute quantification in shotgun proteomics with label-free methods. J Proteomics 73, 1740–1746 (2010). 20576481 10.1016/j.jprot.2010.05.011

[b58] Meisel L. . A rapid and efficient method for purifying high quality total RNA from peaches (Prunus persica) for functional genomics analyses. Biol Res 38, 83–88 (2005). 15977413 10.4067/s0716-97602005000100010

[b59] Rey M. . Mass spec studio for integrative structural biology. Structure 22, 1538–1548 (2014). 25242457 10.1016/j.str.2014.08.013 PMC4391204

