# Author Correction: Addressing proteolytic efficiency in enzymatic degradation therapy for celiac disease

**DOI:** 10.1038/s41598-023-28196-w

**Published:** 2023-01-18

**Authors:** Martial Rey, Menglin Yang, Linda Lee, Ye Zhang, Joey G. Sheff, Christoph W. Sensen, Hynek Mrazek, Petr Halada, Petr Man, Justin L. McCarville, Elena F. Verdu, David C. Schriemer

**Affiliations:** 1 Department of Biochemistry and Molecular Biology and the Southern Alberta Cancer Research Institute, University of Calgary, Calgary, AB Canada; 2 Structural Mass Spectrometry and Proteomics Unit, Institut Pasteur, CNRS UMR 3528, Paris, France; 3 Graz University of Technology, Institute of Molecular Biotechnology, Graz, Austria; 4 Institute of Microbiology, Academy of Sciences of the Czech Republic, and Department of Biochemistry, Faculty of Science, Charles University in Prague, Prague, Czech Republic; 5 Farncombe Family Digestive Health Research Institute, McMaster University, Hamilton, ON Canada

Correction to: *Scientific Reports* https://doi.org/10.1038/srep30980, published online 02 August 2016

This Article contains an error in Figure 6 where the bottom two images are vertical mirror images of each other.

The correct Figure 6 and accompanying legend appear below.

**Figure 6:**
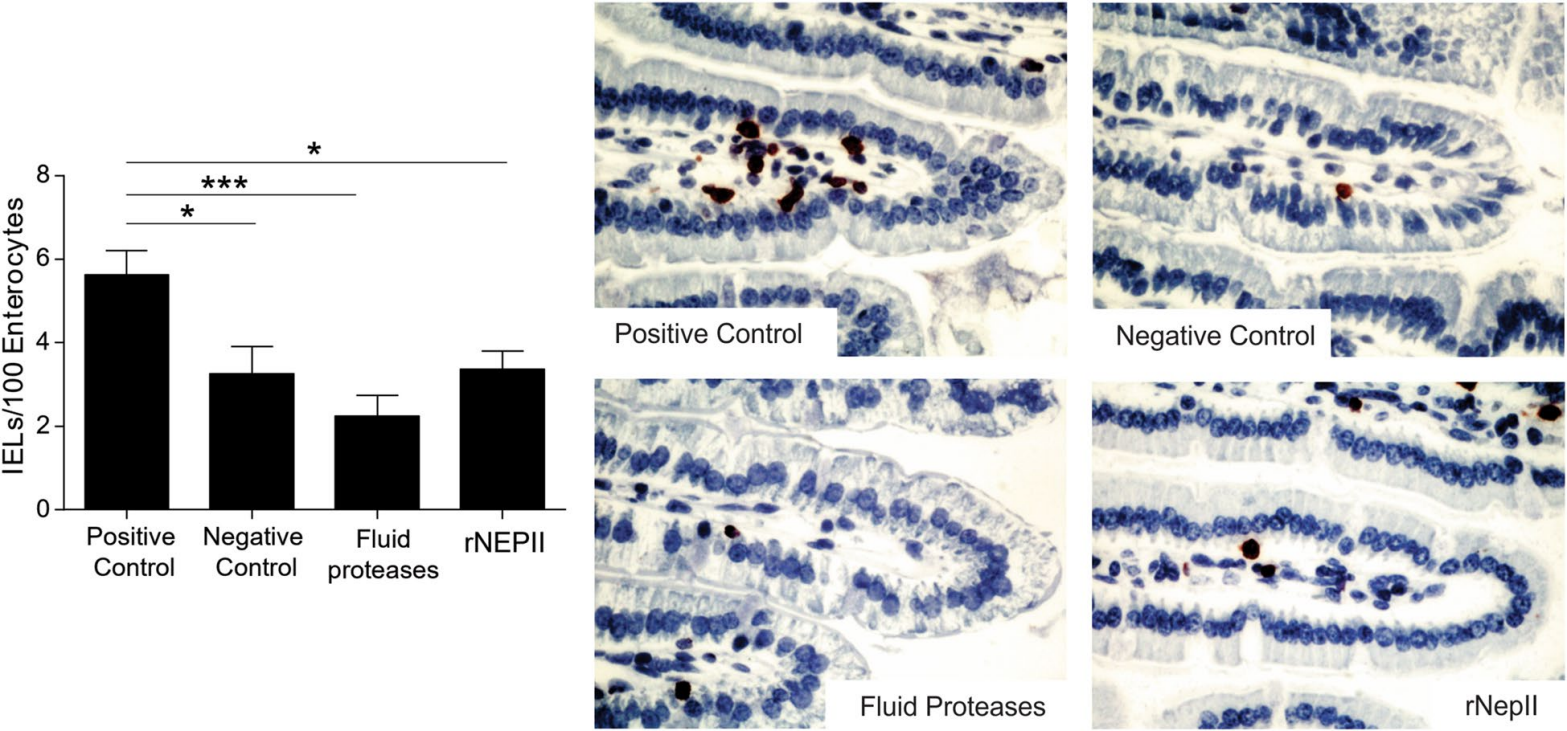
*Nepenthes* fluid protease treatment of gliadin prevents gliadin-induced inflammation in the NOD/DQ8 transgenic mouse model of gluten-sensitivity. Intraepithelial lymphocyte (IEL) counts quantitated by immunostaining of proximal small intestine villi tips, in sensitized mice challenged with pepsin-treated gliadin (positive control), vehicle alone (negative control, gliadin-free diet), fluid protease-treated gliadin (1:264 ratio) and recombinant nepenthesin-II treated gliadin (1:100 ratio). Representative immunostained sections of intestinal tissues shown to the right at 40× magnification. Statistical significance (n = 8 for each state, *p < 0.05, ***p < 0.001).

This change does not affect the conclusions of the Article.

