# Supplementary material for: Addressing proteolytic efficiency in enzymatic degradation therapy for celiac disease
Source: Sci Rep. 2016 Aug 2;6:30980. doi: 10.1038/srep30980 (PMC4969619; doi:10.1038/srep30980)
Supplement: Supplementary Information [file srep30980-s1.pdf]

Supplementary Information

Addressing proteolytic efficiency in enzymatic degradation therapy for celiac disease

Martial Rey, Menglin Yang, Linda Lee, Ye Zhang, Joey G. Sheff, Christoph W. Sensen, Hynek Mrazek, Petr Halada, Petr Man, Justin L McCarville, Elena F. Verdu, David C. Schriemer*

Affiliations:

Farncombe Family Digestive Health Research Institute, McMaster University, Hamilton, ON, Canada
Justin L McCarville, Elena F. Verdu

Institute of Microbiology, Academy of Sciences of the Czech Republic, and Department of Biochemistry, Faculty of Science, Charles University in Prague, Prague, Czech Republic
Hynek Mrazek, Petr Halada, Petr Man

Graz University of Technology, Institute of Molecular Biotechnology, Graz, Austria
Christoph W. Sensen

Structural Mass Spectrometry and Proteomics Unit, Institut Pasteur, CNRS UMR 3528, Paris, France
Martial Rey

Department of Biochemistry and Molecular Biology and the Southern Alberta Cancer Research Institute, University of Calgary, Calgary, Alberta, Canada
Martial Rey, Menglin Yang, Linda Lee, Ye Zhang, Joey G. Sheff, David C. Schriemer

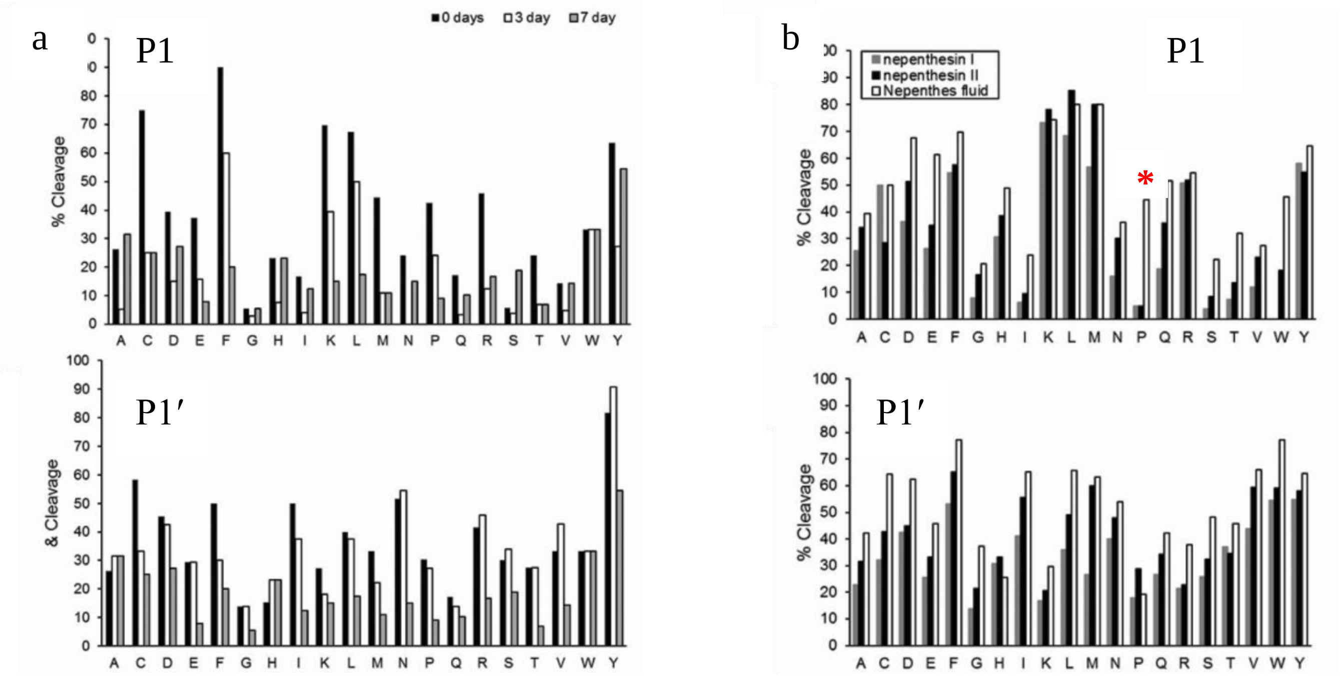

Supplementary Figure S1. (a) Characterization and stability testing of protein concentrate from stimulated *Nepenthes* fluid. C-terminal cleavage preferences (P1 position, top) and N-terminal cleavage preferences (P1' position, bottom) as a function of storage time at elevated temperature. Fluid concentrate was incubated at 37 ˚C for 0-7 days. No alteration of the broadly non-specific digestion character of the extract is observed over time. The global reduction correlates with a modest overall reduction in total activity as measured using the hemoglobin assay (not shown). Data was collected from 2-minute in-solution digestions of protein standards at 37 ˚C (pH 2.5), and cleavage preferences were determined using LC-MS/MS methods. For each amino acid, cleavage preferences were estimated by calculating the number observed terminal residues relative to the total number of residues, in percent. **(b) Cleavage preferences of recombinant nepenthesin I and II compared to *Nepenthes* fluid protein concentrate.** Cleavage of residues in the P1 position (top) and in the P1' positions (bottom) are shown as relative % cleavage to the total, as defined above, using LC-MS/MS data from a set of protein standards. Recombinant aspartic proteases were prepared as described previously (1-3). Samples were digested in solution for 5 minutes at 37 ˚C in this comparison, for all three enzyme preparations. The absence of C-terminal proline cleavage (red asterisk) represents the major difference between recombinant enzymes and the fluid protein concentrate.

1. Yang M*, et al.* (2015) Recombinant Nepenthesin II for Hydrogen/Deuterium Exchange Mass Spectrometry. (Translated from eng) *Anal Chem* 87(13):6681-6687 (in eng).
2. Kadek A*, et al.* (2014) Expression and characterization of plant aspartic protease nepenthesin-1 from Nepenthes gracilis. (Translated from eng) *Protein Expr Purif* 95:121-128 (in eng).
3. Kadek A*, et al.* (2014) Aspartic protease nepenthesin-1 as a tool for digestion in hydrogen/deuterium exchange mass spectrometry. (Translated from eng) *Anal Chem* 86(9):4287-4294 (in eng).

a

```
  1 MQAKFFTFVI LSSVFYFNYP LAEARSIQAR LANKPKGTIK TIKGDDGEVV
 51 DCVDIYKQPA FDHPLLKNHT LQMQPSSYAS KVGEYNKLEQ PWHKNGECPK
101 GSIPIRRQVI TGLPVVKKQF PNLKFAPPSA NTNHQYAVIA YFYGNASLQG
151 ANATINIWEP NLKNPNGDFS LTQIWISAGS GSSLNTIEAG WQVYPGRTGD
201 SQPRFFIYWT ADGYTSTGCY DLTCPGFVQT NNYYAIGMAL QPSVYGGQQY
251 ELNESIQRDP ATGNWWLYLW GTVVGYWPAS IYNSITNGAD TVEWGGEIYD
301 SSGTGGFHTT TQMGSGHFPT EGYGKASYVR DLQCVDTYGN VISPTANSFQ
351 GIAPAPNCYN YQFQQGSSEL YLFYGGPGCQ *
```

Signal Peptide - DUF4409 (38-138) -linker- DUF239 (149-380)

MS/MS coverage

Possible start sites for mature enzyme, based on MW analysis of enzyme from fluid and sequence analysis of digested products.

b

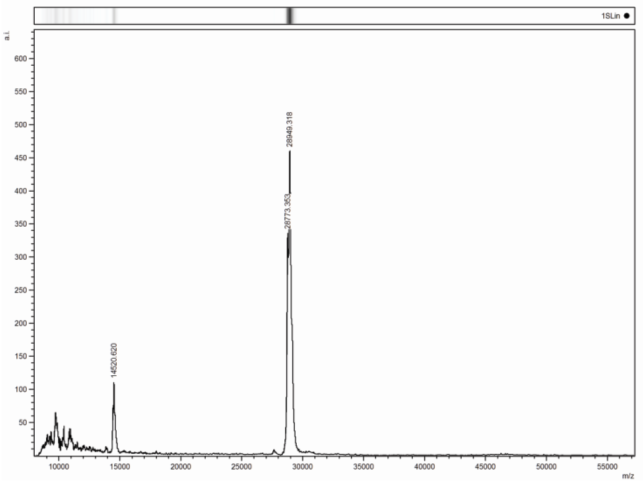

c

Hit	Experimental m/z	Experimental z	Theoretical peptide mass	Delta mass (expt-theor)	Start	Stop	Expectation value (Mascot)	Peptide sequence
1	503.2208	2	1004.4312	-0.0041	129	137	0.0057	SANTNHQYA
2	552.7559	2	1103.4996	-0.0023	129	138	0.00012	SANTNHQYAV
3	609.297	2	1216.5836	-0.0042	129	139	9.50E-05	SANTNHQYAVI
4	644.8159	2	1287.6207	-0.0035	129	140	0.0006	SANTNHQYAVIA
5	726.3444	2	1450.6841	-0.0098	129	141	0.0028	SANTNHQYAVIAY
6	698.8416	2	1395.6783	-0.0096	157	168	1.50E-05	IWEPNLKNPNGD
7	772.3779	2	1542.7467	-0.0054	157	169	5.10E-05	IWEPNLKNPNGDF
8	872.4329	2	1742.8628	-0.0115	157	171	0.00057	IWEPNLKNPNGDFSL
9	509.7482	2	1017.4879	-0.0061	161	169	0.032	NLKNPNGDF
10	539.2798	2	1076.5502	-0.0052	174	184	2.00E-08	IWISAGSGSSL
11	482.737	2	963.4662	-0.0067	175	184	3.30E-06	WISAGSGSSL
12	590.2819	2	1178.5568	-0.0075	175	186	3.60E-07	WISAGSGSSLNT
13	497.2435	2	992.4774	-0.005	176	186	7.30E-06	ISAGSGSSLNT
14	722.0198	3	2163.0498	-0.0122	187	205	0.0016	IEAGWQVYPGRTGDSQPRF
15	961.4634	2	1920.9231	-0.0109	189	205	0.00014	AGWQVYPGRTGDSQPRF
16	598.6247	3	1792.8645	-0.0123	191	205	0.017	WQVYPGRTGDSQPRF
17	652.8132	2	1303.6157	-0.0038	192	203	0.0052	QVYPGRTGDSQP
18	804.3984	2	1606.7852	-0.003	192	205	0.00098	QVYPGRTGDSQPRF
19	560.7759	2	1119.5421	-0.0049	196	205	0.032	GRTGDSQPRF
20	568.2549	2	1134.5022	-0.007	206	214	0.001	FIYWTADGY
21	792.835	2	1583.6603	-0.0048	206	219	0.00016	FIYWTADGYTSTGC
22	520.2419	2	1038.4771	-0.0078	225	233	0.00028	PGFVQTNNY
23	639.8085	2	1277.6074	-0.005	238	249	5.10E-07	MALQPSVYGGQQ
24	574.2883	2	1146.5669	-0.0049	239	249	8.50E-05	ALQPSVYGGQQ
25	538.77	2	1075.5298	-0.0044	240	249	1.20E-05	LQPSVYGGQQ
26	744.3438	2	1486.6801	-0.007	253	265	7.90E-06	NESIQRDPATGNW
27	622.8029	2	1243.5945	-0.0033	255	265	2.40E-06	SIQRDPATGNW
28	715.8408	2	1429.6739	-0.0068	255	266	2.60E-06	SIQRDPATGNWW
29	772.3814	2	1542.7579	-0.0097	255	267	0.00012	SIQRDPATGNWWL
30	579.2859	2	1156.5625	-0.0053	256	265	1.30E-06	IQRDPATGNW
31	672.3248	2	1342.6418	-0.0068	256	266	6.50E-06	IQRDPATGNWW
32	568.2799	2	1134.5499	-0.0046	270	279	6.10E-06	WGTVVGYWPA
33	611.7955	2	1221.5819	-0.0054	270	280	3.40E-05	WGTVVGYWPAS
34	668.3383	2	1334.6659	-0.0039	270	281	1.70E-05	WGTVVGYWPASI
35	546.7875	2	1091.5652	-0.0047	272	281	0.00019	TVVGYWPASI
36	577.7667	2	1153.5251	-0.0063	280	290	1.10E-05	SIYNSITNGAD
37	534.2499	2	1066.4931	-0.0078	281	290	0.00048	IYNSITNGAD
38	477.7091	2	953.409	-0.0054	282	290	0.0015	YNSITNGAD
39	584.7576	2	1167.5084	-0.0078	291	300	0.0018	TVEWGGEIYD
40	881.3793	2	1760.753	-0.0089	291	307	1.20E-10	TVEWGGEIYDSSGTGGF
41	716.8011	2	1431.5943	-0.0067	294	307	3.70E-10	WGGEIYDSSGTGGF
42	502.2184	2	1002.4295	-0.0072	298	307	3.90E-08	IYDSSGTGGF
43	786.3497	2	1570.69	-0.0052	298	312	1.30E-06	IYDSSGTGGFHTTTQ
44	315.6812	2	629.3497	-0.0018	329	333	0.033	VRDLQ
45	367.1854	2	732.3589	-0.0026	329	334	0.089	VRDLQC
46	367.1857	2	732.3589	-0.002	329	334	0.011	VRDLQC
47	474.2316	2	946.4542	-0.0056	329	336	0.024	VRDLQCVD
48	532.7635	2	1063.5186	-0.0062	335	344	5.60E-05	VDTYGNVISP
49	792.8765	2	1583.7468	-0.0083	335	349	3.30E-07	VDTYGNVISPTANSF
50	587.2949	2	1172.5826	-0.0073	345	356	1.50E-05	TANSFQGIAPAP
51	695.8201	2	1389.6347	-0.009	345	358	0.00092	TANSFQGIAPAPNC
52	777.3538	2	1552.698	-0.005	345	359	2.20E-08	TANSFQGIAPAPNCY
53	834.3742	2	1666.7409	-0.0071	345	360	8.20E-09	TANSFQGIAPAPNCYN
54	915.9063	2	1829.8043	-0.0062	345	361	0.0085	TANSFQGIAPAPNCYNY
55	435.7085	2	869.4065	-0.0041	350	358	0.0053	QGIAPAPNC
56	517.2383	2	1032.4698	-0.0078	350	359	4.10E-05	QGIAPAPNCY
57	574.2609	2	1146.5128	-0.0055	350	360	1.60E-05	QGIAPAPNCYN
58	594.2492	2	1186.4891	-0.0052	360	369	3.50E-07	NYQFQQGSSE

Supplementary Figure S2. Mass analysis supports the identification of neprosin in the fraction isolated from the fluid extract, based on the gene sequence discovered by RNA-seq. (a) Protein sequence for neprosin, identified from a combination of RNA-seq data, *de novo* peptide sequencing from nonspecific digests of the fraction analyzed by LC-MS/MS, and extended to full length using 5′ and 3′ RACE. Domain boundaries based on designations in Pfam, and signal peptide detected by SignalP4.1. (b) MALDI-TOF analysis of the protein content of the isolated fraction confirms purity and suggests a mature enzyme is smaller than the full sequence in A. Estimated molecular weight is 28,860. (c) Peptides identified from a non-specific (peptic) digestion of isolated, denatured neprosin, supporting the sequence identified using 5′ and 3′ RACE (51% coverage). Output represents the unique peptides detected using Mascot v2.3, filtered for p<0.05.

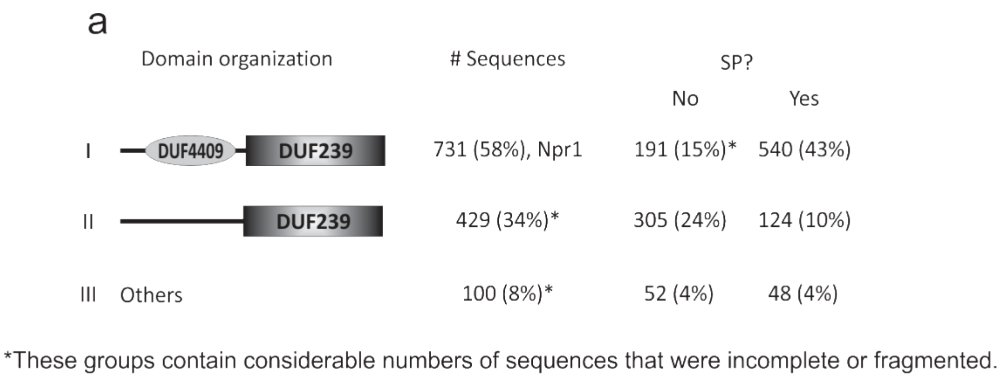

b

	Accession ID	Species	% Identity
1	Npr1_Nven	*Nepenthes ventrata*	100
2	XP_010923368.1	*Elaeis guineensis*	46
3	BAD68526.1	*Oryza sativa japonica*	47
4	XP_008677862.1	*Zea mays*	45
5	XP_004979373.1	*Setaria italica*	47
6	EMT22075.1	*Aegilops tauschii*	47
7	ERM98017.1	*Amborella trichopoda*	46
8	XP_010523377.1	*Tarenaya hassleriana*	46
9	XP_013690431.1	*Brassica napus*	47
10	XP_006293539.1	*Capsella rubella*	46
11	AAX55164.1	*Arabidopsis thaliana*	47
12	XP_010508321.1	*Camelina sativa*	47

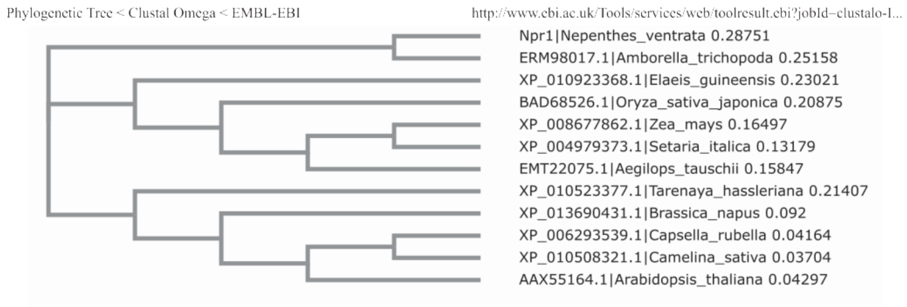

Supplementary Figure S3. Neprosin represents a newly discovered enzyme functionality. (a) Domain organization of Npr and other DUF239 family members (Pf3080). Most entries in the Pfam database contain DUF4409 and DUF239 in tandem, and most possess a signal peptide. The category of "other" includes various domain repeats and DUF4409 alone. (b) top: BLAST hits with the highest percent identity to Npr1. Bottom: hierarchical clustering based on ClustalW sequence alignments.

Protease family	Name	Species	# amino acids	Identity (%)[a]	Substrate specificity	Domain organization[b]
DUF239	Neprosin (Npr1)	*Nepenthes ventrata*	380	100	Pro-X	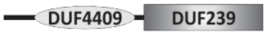
	DUF4409-DUF239[c]	*Brassica napus*	402	47		
S9A	Prolyl oligopeptidase (POP)	*Pyrococcus furiosus*	616	<20	C-terminal Pro-X oligopeptide	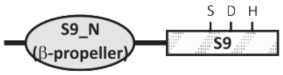
		Myxococcus xanthus	692	<20		
		Flavobacterium meningosepticum	705	<20		
		Sphingomonas capsulate	723	<20		
S28	Prolyl endoprotease (PEP)	*Aspergillus niger*	526	<20	Pro-X	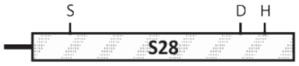
S9B	Dipeptidyl-peptidase IV (DPP4)	*Aspergillus oryzae*	771	<20	N-terminal Pro-X dipeptide	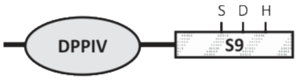
M24B	Aminopeptidase P (APP)	*Sus scrofa*	673	<20	N-terminal X-Pro dipeptide	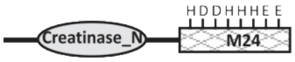

[a]identity referenced to the sequence of neprosin
[b]domain organization from Pfam, with active site residue highlighted
[c]association of this entry to Pro-X functionality is speculative

Supplementary Figure S4. Neprosin represents a newly discovered enzyme functionality. Comparison between neprosin and known proline-cleaving enzymes that have bee studied for gluten detoxification, highlighting either sequence, functional or structural dissimilarity with known proline-cleaving enzymes.

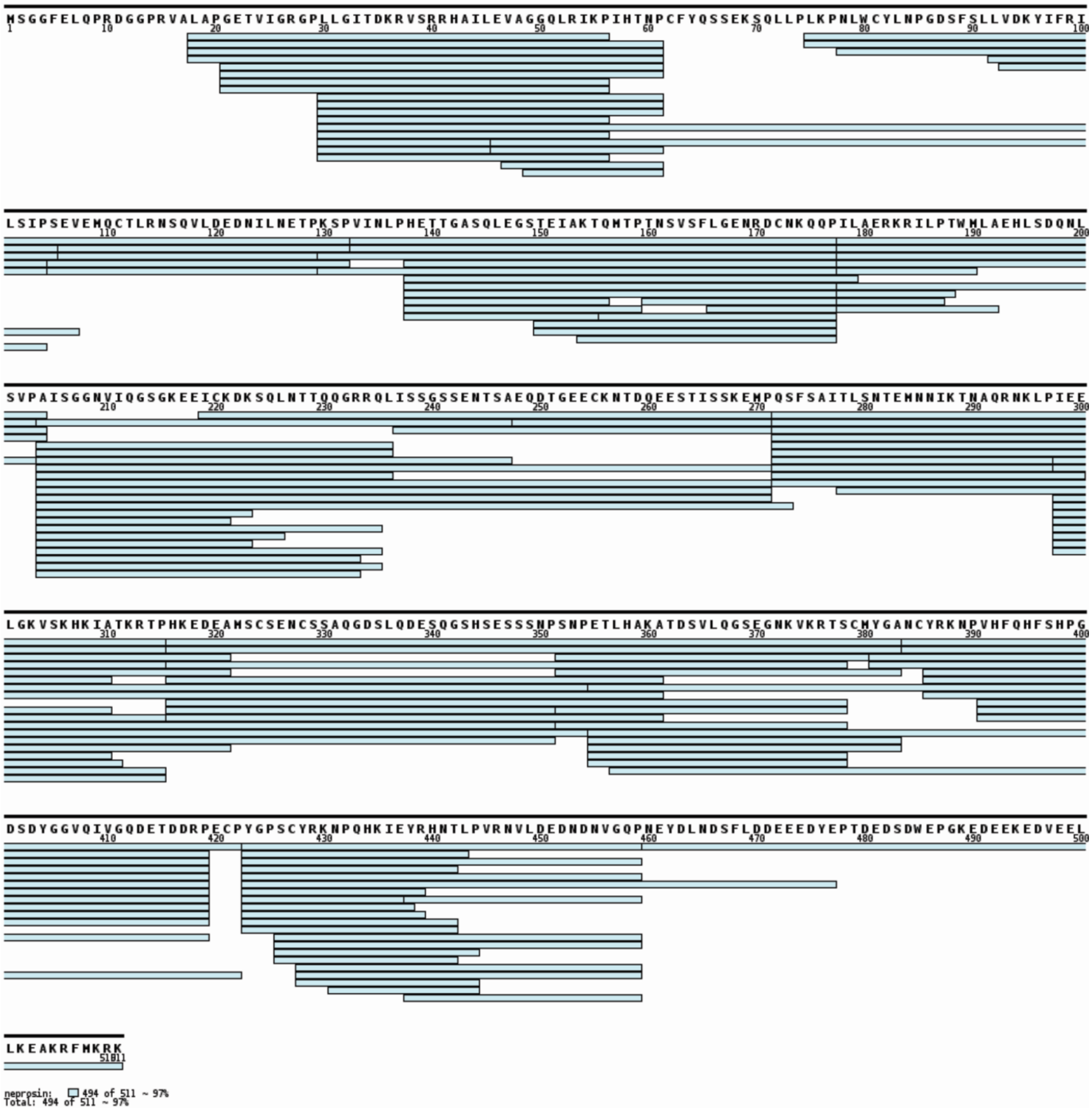

Supplementary Figure S5. Data-dependent LC-MS/MS analysis of APLF digested with Neprosin. A sequence map is shown for the protein "Aprataxin and PNK-Like Factor" (APLF), a 511 residue protein with a moderately high frequency of proline residues. Data was generated using HCD fragmentation on an LC-Orbitrap Velos instrument, and data searched against the sequence of APLF in Mass Spec Studio assuming no enzyme specificity, and results were cut off at a peptide false discovery rate (FDR) of 0.5%.

MS/MS-based quantitation of digestion products:

The MS/MS peptide fragmentation data supports an identification of digestion products, and an alternative quantitative analysis. Based on a database search comprised of known gluten proteins, we confirmed that the crude gliadin fraction contains a distribution of α/β-gliadin and γ-gliadin isotypes. Many peptides were also evident for the glutenins. The low molecular weight subunit is particularly abundant, highlighting the crude nature of the conventional gliadin extraction process (Table S4). The weighted average peptide length for the 5 μM pepsin digest was 16.9 residues (based on 1030 features), which accounted for only 13% of the total digest signal. The fluid protease digestion at 0.46 μM produced an average peptide length of 11.2 residues (1370 features), accounting for 30% of the total signal. Co-digestion using these two concentrations generated a weighted average of 10.2 residues (1571 features) accounting for 40% of the digest signal. This fraction represents a high signal usage rate in a proteomic experiment[1]. That is, the unidentified fraction mostly represents a combination of sampling rate limitations and insignificant peptide scores, rather than undigested protein. Longer digestions using the combined proteases reduces total LC-MS signal without changing the size distribution, consistent with a proteomics method that cannot detect peptides < 6 amino acid residues in length. Taken together, the proteomics data point to an extensive digestion of crude gliadin under the action of low-concentration fluid proteases, where co-digestion with pepsin enhances proteolysis even when fluid enzymes appear saturating.

1. Chick, J.M. et al. A mass-tolerant database search identifies a large proportion of unassigned spectra in shotgun proteomics as modified peptides. *Nat Biotechnol* 33, 743-749 (2015).

a

Protein sequence coverage: 76% (88 cleavage sites detected)

1 MKTFLILALL AIVATTARIA VRVPVPQLQP QNPSQQQPQE QVPLVQQQQF

51 PGQQQPFPPQ QPYPQPQPFP SQQPYLQLQP FPQPQLPYPQ PQLPYPQPQL

101 PYPQPQPFRP QQPYPQSQPQ YSQPQQPISQ QQQQQQQQQQ QKQQQQQQQQ

151 ILQQILQQQL IPCRDVVLQQ HSIAYGSSQV LQQSTYQLVQ QLCCQQLWQI

201 PEQSRCQAIH NVVHAIILHQ QQQQQQQQQQ QPLSQVSFQQ PQQQYPSGQG

251 SFQPSQQNPQ AQGSVQPQQL PQFEEIRNLA LETLPAMCNV YIPPYCTIAP

301 VGIFGTN

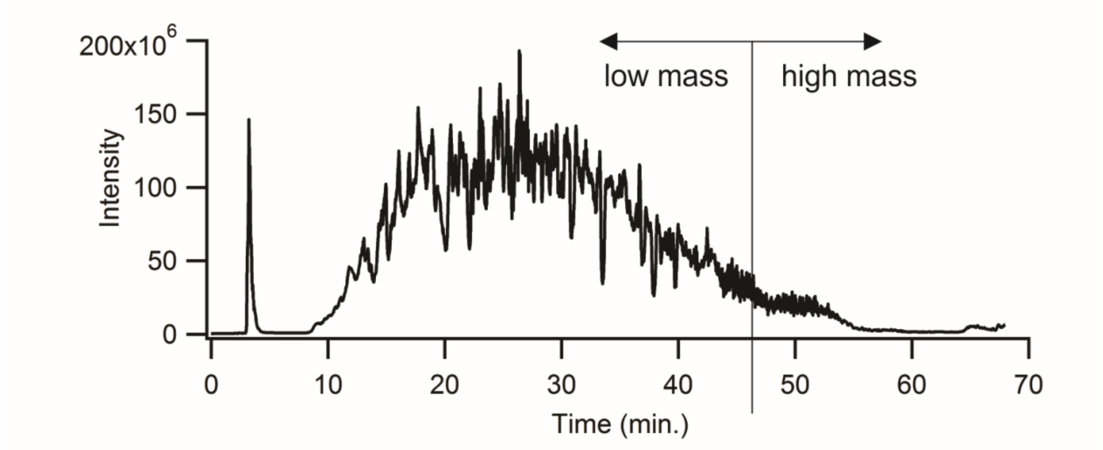

b

Protein sequence coverage: 49% (40 cleavage sites detected)

1 MKTFLILALL AIVATTARIA VRVPVPQLQP QNPSQQQPQE QVPLVQQQQF

51 PGQQQPFPPQ QPYPQPQPFP SQQPYLQLQP FPQPQLPYPQ PQLPYPQPQL

101 PYPQPQPFRP QQPYPQSQPQ YSQPQQPISQ QQQQQQQQQQ QKQQQQQQQQ

151 ILQQILQQQL IPCRDVVLQQ HSIAYGSSQV LQQSTYQLVQ QLCCQQLWQI

201 PEQSRCQAIH NVVHAIILHQ QQQQQQQQQQ QPLSQVSFQQ PQQQYPSGQG

251 SFQPSQQNPQ AQGSVQPQQL PQFEEIRNLA LETLPAMCNV YIPPYCTIAP

301 VGIFGTN

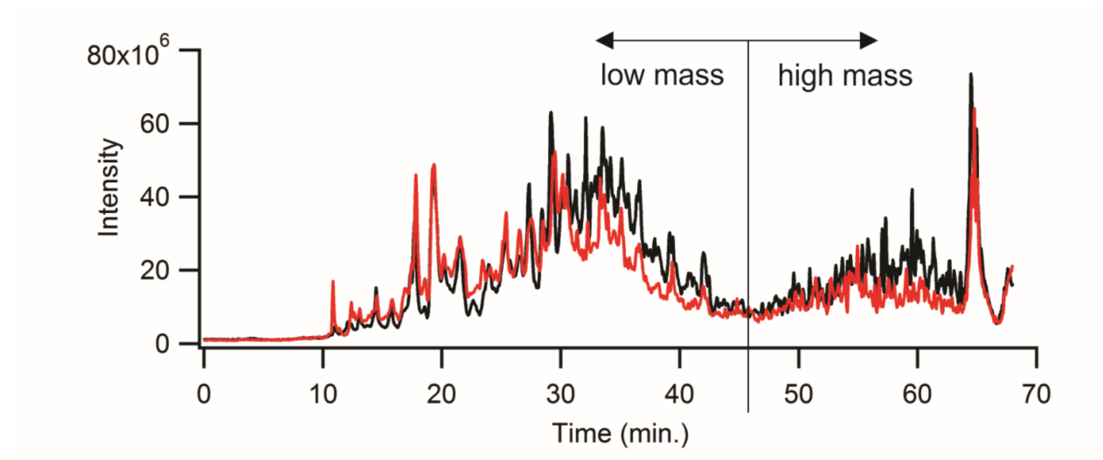

Supplementary Figure S6. Neprosin only partially reconstitutes the gliadin digestion efficiency. Sequence coverage map for α/β-gliadin MM1 (Uniprot P18573.1) in crude gliadin, and associated total ion chromatograms. (a) Digestion used 0.46 μM fluid protease for 90 min at 37 ºC. This enzyme concentration is the same used in the study (see Figure 4). (b) Black trace represents a neprosin concentration matched to the highest level tested in the fluid extract in A (~0.1 μM) and the red trace represents double this concentration (~0.2 μM). For both (a) and (b), sequence coverage is highlighted using red text, with cleavage sites marked in bold cyan. The chromatograms are marked with approximate boundaries for the sizes of digest products.

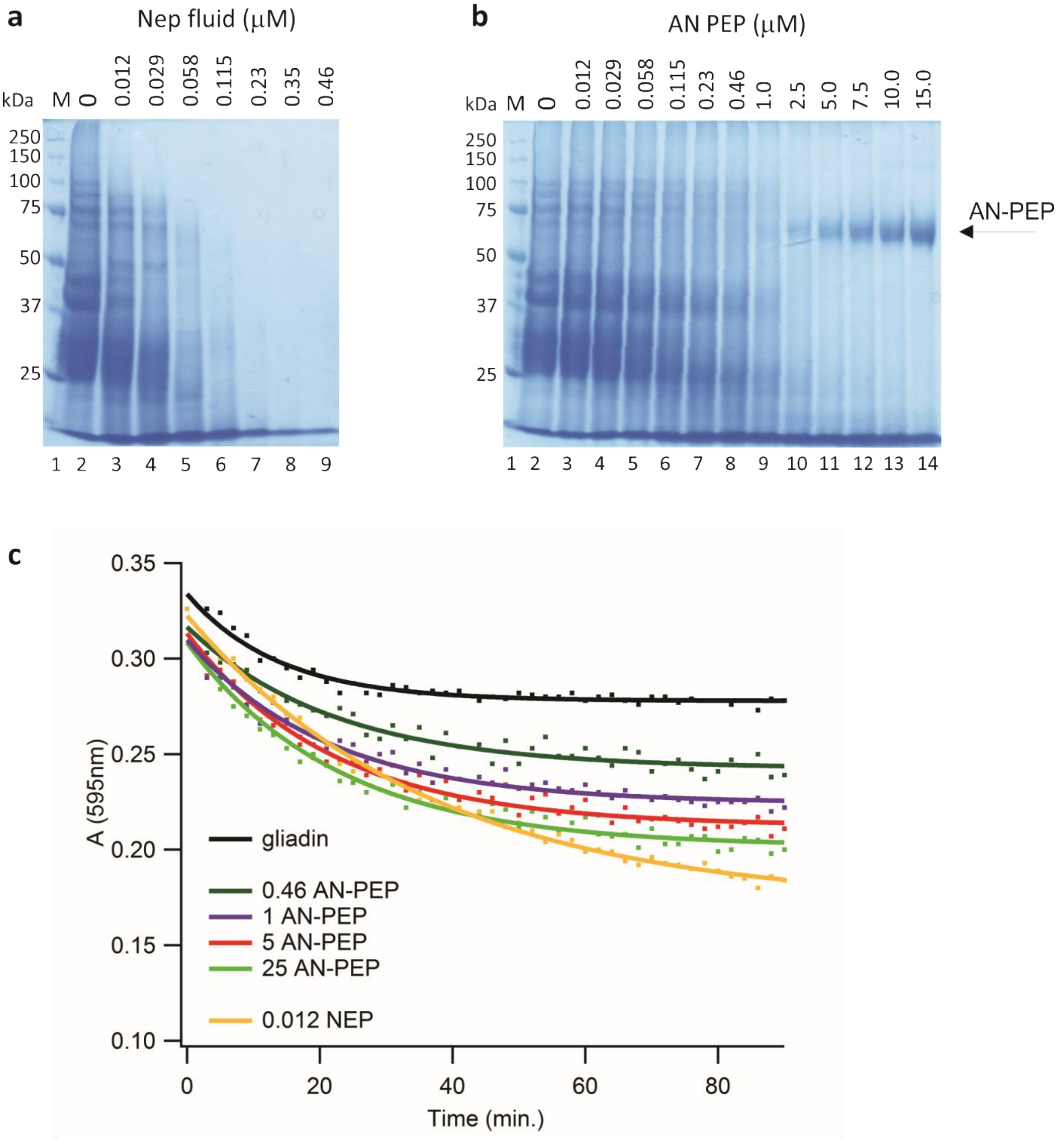

Supplementary Figure S7. Comparison of digestion characteristics between *Nepenthes* enzymes (fluid extract) and AN-PEP. (a) Gel re-analysis of crude gliadin slurry with *Nepenthes* enzymes, as in Figure 4. (b) Corresponding gel analysis of crude gliadin slurry using AN-PEP. (c) Turbidometric timecourse analysis of digestion using crude gliadin slurry, as in Figure 3, where the enzyme concentrations are indicated in units of micromolar. NEP: *Nepenthes* enzyme extract. AN-PEP: *Aspergillus niger* prolyl endoprotease.

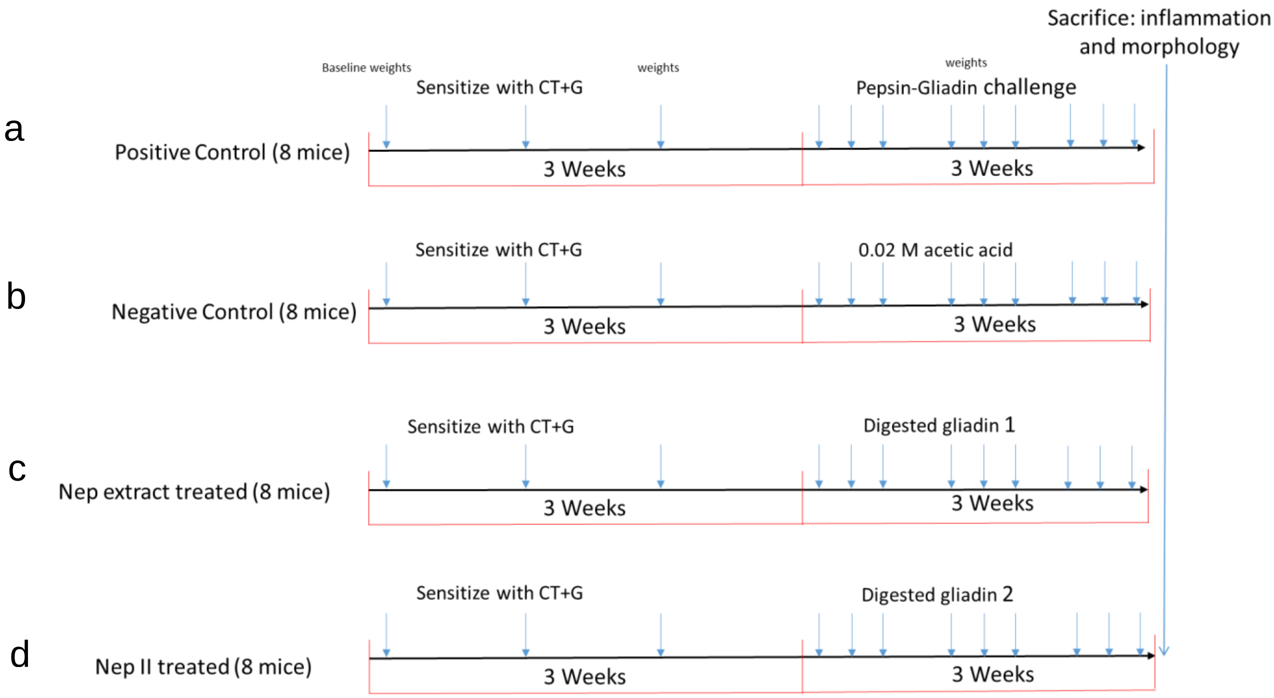

Supplementary Figure S8. Experiment design and gliadin feeding schedule of NOD DQ8 transgenic mice. 4x8 groups of mice were sensitized using cholera toxin (CT) and pepsin-gliadin (P-G) once per week for three weeks. P-G was prepared with 100:1 wt. ratio of gliadin to enzyme. Each group was then challenged three times per week for three weeks with (a) P-G doses as a positive control for intestinal inflammation (b) 0.02M acetic acid vehicle (c) gliadin codigested with pepsin at a 100:1 ratio and with fluid enzyme concentrate at a 264:1 ratio (d) gliadin codigested with pepsin at a 100:1 ratio and with nepenthesin II at a 100:1 ratio. All doses were prepared in 5 mg quantities, digested for 90 min. at 37ºC then lyophilized. Dried feed was reconstituted using 0.02M acetic acid at dosing.

Supplementary Table S1: Gel-free bottom-up proteome analysis of *Nepenthes* pitcher fluid, digested with trypsin.

Rank	accession number	Protein name	Taxonomy	# of peptides*
1	gi|38325811	Heat-shock protein 70-1	*N. tabacum*	5 (47)
2	gi|326499079	predicted protein	*H. vulgare*	8 (49)
**3**	**gi|61214233**	**nepenthesin-1**	***N. gracilis***	**1 (36)**
4	gi|326492680	predicted protein	*H. vulgare*	6 (52)
5	gi|308044587	Uncharacterized protein LOC100501669	*Zea Mays*	4 (26)
6	gi|226532205	Uncharacterized protein LOC100274495	*Zea Mays*	2 (19)
7	gi|226510502	Uncharacterized protein LOC100273100	*Zea Mays*	8 (38)
8	gi|326502504	Predicted protein	*H. vulgare*	2 (18)
9	gi|226507094	Uncharacterized protein LOC100273141	*Zea Mays*	2 (15)
10	gi|326502344	Predicted protein	*H. vulgare*	1 (8)

*The total number of unique peptides identified (total number of peptides in brackets). Ion cut-off score ($p<0.05$): 40

Supplementary Table S2. Gel-free bottom-up proteome analysis of deglycosylated *Nepenthes* pitcher fluid, digested with trypsin.

Rank	accession number	Protein name	Taxonomy	# of peptides*
1	gi|165292442	class IV chitinase	*N. alata*	4 (26)
2	gi|85682819	thaumatin-like protein	*N. gracilis*	2 (20)
**3**	**gi|61214233**	**Nepenthesin-1**	***N. gracilis***	**1 (31)**
4	gi|393387669	β-1,3-glucanase	*N. alata*	3 (28)
5	gi|167998797	Predicted protein	*P. pattens*	1 (7)
6	gi|294461233	unknown	*Picea sitchensis*	1 (41)
7	gi|413924608	Hypothetical protein	*Zea Mays*	1 (96)
8	gi|527192719	Hypothetical protein	*Genlisea aurea*	1 (69)
9	gi|205830697	Unknown protein 18	*P. menziesii*	1 (2)
10	gi|2493495	Serine-carboxypeptidase	*Pisum sativum*	1 (6)

*The total number of unique peptides identified (total number of peptides in brackets). Ion cut-off score ($p<0.05$): 39

Supplementary Table S3. Gel-free bottom-up proteome analysis of *Nepenthes* pitcher fluid, digested with active *Nepenthes* pitcher fluid.

Rank	accession number	Protein name	Taxonomy	# of peptides*
**1**	**gi|409179880**	**Nepenthesin II**	***Nepenthes mirabilis***	**40 (143)**
**2**	**gi|61214233**	**Nepenthesin I**	***Nepenthes gracilis***	**16 (57)**
3	gi|218201535	Hypothetical protein	*Oryza sativa*	1 (1)
4	gi|508715246	ARM repeat superfamily protein	*Theobroma cacao*	1 (1)
5	gi|502179750	Predicted, uncharacterized protein	*Cicer arietinum*	1 (1)
6	gi|502146217	Predicted, acid phosphatase-like protein	*Cicer arietinum*	1 (1)
7	gi|255549692	Hypothetical protein	*Ricinus communis*	1 (1)

*The total number of unique peptides identified (total number of peptides in brackets)
Ion cut-off score ($p<0.05$): 60

Supplementary Table S4. Proteomic characterization of crude gliadin preparation

#	Accession #	Mascot Protein Score	Mass (Da)	Matches[a]	Sequences[a]	emPAI[b]	Name
1	gi|121094|sp|P04725.1|GDA5_WHEAT	10424	36643	489 (450)	116 (110)	36883.01	RecName: Full=Alpha/beta-gliadin A-V; AltName: Full=Prolamin; Flags: Precursor
2	gi|121098|sp|P18573.1|GDA9_WHEAT	9282	35375	425 (391)	124 (119)	128026.67	RecName: Full=Alpha/beta-gliadin MM1; AltName: Full=Prolamin; Flags: Precursor
3	gi|121096|sp|P04727.1|GDA7_WHEAT	8302	36095	409 (372)	107 (103)	19651.69	RecName: Full=Alpha/beta-gliadin clone PW8142; AltName: Full=Prolamin; Flags: Precursor
4	gi|121093|sp|P04724.1|GDA4_WHEAT	8238	34217	355 (316)	102 (96)	13300.51	RecName: Full=Alpha/beta-gliadin A-IV; AltName: Full=Prolamin; Flags: Precursor
5	gi|121101|sp|P08453.1|GDB2_WHEAT	8194	37099	523 (424)	116 (113)	15151.72	RecName: Full=Gamma-gliadin; Flags: Precursor
6	gi|121457|sp|P10386.1|GLTB_WHEAT	8085	34906	455 (425)	127 (122)	74512.08	RecName: Full=Glutenin, low molecular weight subunit 1D1; Flags: Precursor
7	gi|121104|sp|P21292.1|GDBX_WHEAT	7655	34278	447 (361)	106 (99)	4284.08	RecName: Full=Gamma-gliadin; Flags: Precursor
8	gi|121095|sp|P04726.1|GDA6_WHEAT	7417	33920	377 (332)	83 (80)	3201.36	RecName: Full=Alpha/beta-gliadin clone PW1215; AltName: Full=Prolamin; Flags: Precursor
9	gi|121091|sp|P04722.1|GDA2_WHEAT	6989	33640	345 (306)	110 (101)	35998.64	RecName: Full=Alpha/beta-gliadin A-II; AltName: Full=Prolamin; Flags: Precursor
10	gi|121090|sp|P04721.1|GDA1_WHEAT	6564	30384	309 (281)	103 (98)	51350.05	RecName: Full=Alpha/beta-gliadin A-I; AltName: Full=Prolamin; Flags: Precursor
11	gi|121103|sp|P06659.1|GDBB_WHEAT	6199	32946	506 (391)	110 (101)	12781.88	RecName: Full=Gamma-gliadin B; Flags: Precursor
12	gi|67464993|sp|P02863.2|GDA0_WHEAT	6117	32943	284 (250)	98 (93)	8722.77	RecName: Full=Alpha/beta-gliadin; AltName: Full=Prolamin; Flags: Precursor
13	gi|121099|sp|P08079.1|GDB0_WHEAT	5659	29035	457 (344)	108 (93)	13280.46	RecName: Full=Gamma-gliadin; Flags: Precursor
14	gi|121102|sp|P04730.1|GDB3_WHEAT	5003	27320	270 (258)	73 (69)	2435.24	RecName: Full=Gamma-gliadin; AltName: Full=Gliadin B-III
15	gi|121455|sp|P10385.1|GLTA_WHEAT	4238	40994	407 (318)	89 (85)	354.42	RecName: Full=Glutenin, low molecular weight subunit; Flags: Precursor
16	gi|121092|sp|P04723.1|GDA3_WHEAT	3876	32216	185 (179)	67 (63)	698.01	RecName: Full=Alpha/beta-gliadin A-III; AltName: Full=Prolamin; Flags: Precursor
17	gi|121449|sp|P10387.1|GLT0_WHEAT	3592	69587	154 (139)	70 (67)	21.66	RecName: Full=Glutenin, high molecular weight subunit DY10; Flags: Precursor
18	gi|300669719|sp|P10388.5|GLT5_WHEAT	3555	90239	176 (169)	63 (58)	7.72	RecName: Full=Glutenin, high molecular weight subunit DX5; Flags: Precursor
19	gi|121453|sp|P08489.1|GLT4_WHEAT	2819	89120	141 (135)	54 (49)	5.25	RecName: Full=Glutenin, high molecular weight subunit PW212; Flags: Precursor
20	gi|121100|sp|P04729.1|GDB1_WHEAT	2814	34230	239 (188)	70 (64)	480.43	RecName: Full=Gamma-gliadin B-I; Flags: Precursor

21	gi|121459|sp|P16315.1|GLTC_WHEAT	2043	33358	225 (160)	52 (47)	83.02	RecName: Full=Glutenin, low molecular weight subunit PTDUCD1; Flags: Precursor
22	gi|121097|sp|P04728.1|GDA8_WHEAT	1799	21505	62 (61)	32 (31)	86.31	RecName: Full=Alpha/beta-gliadin clone PTO-A10; AltName: Full=Prolamin
23	gi|121450|sp|P02861.1|GLT1_WHEAT	484	10889	29 (28)	9 (8)	7.99	RecName: Full=Glutenin, high molecular weight subunit PC256

[a] First number the total count. Number in brackets the total count above the significance threshold ($p < 0.05$).

[b] Exponentially Modified Protein Abundance Index, providing a label-free relative quantitation of proteins based on protein coverage using the peptides matches in the search result. While based on partially redundant (non-unique) peptide identifications, sufficient numbers of unique peptides are evident for each entry in the table, as supported by the hierarchical cluster-graph below. Here, branch-points represent the cumulative score for significant peptides matches that would have to be discarded to remove any differentiation between branches.

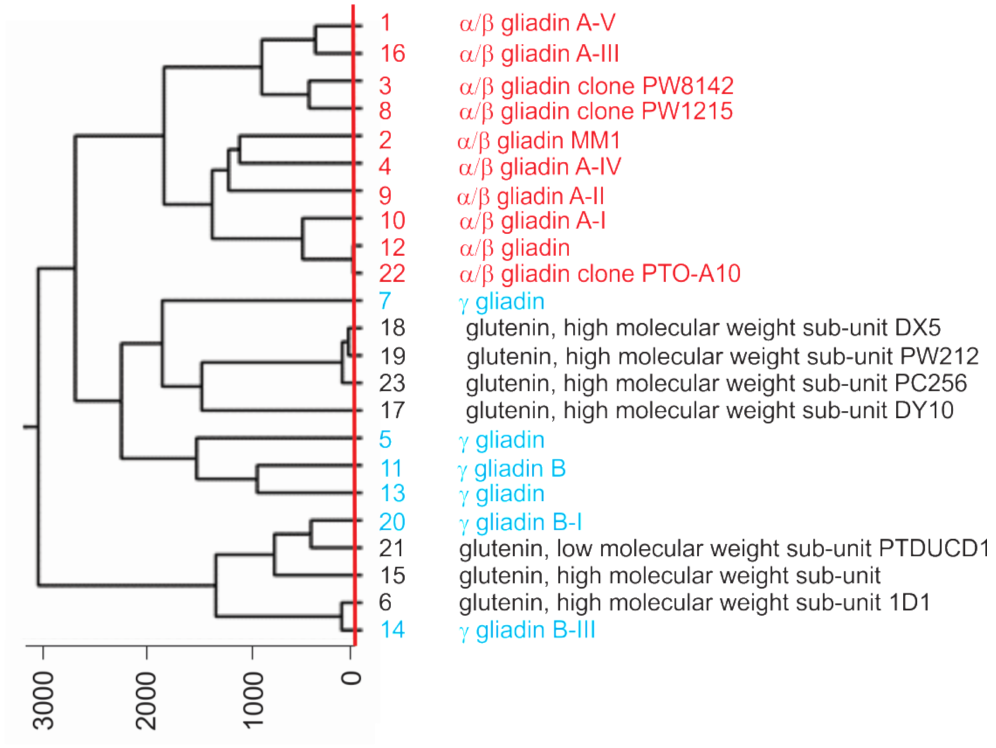

Supplementary Table S5. Analysis of peptide conversion to deamidated forms in total crude gliadin digest, using LC-MS/MS

Nepenthes protease conc. (μM)[a]	Deamidation ratio (antigenic region, wt.)[b]	# LC/MS features	Avg. Length (# AA)	Deamidation ratio (non-antigenic region, wt.)[b]	# LC/MS features	Avg. Length (# AA)	Conversion ratio[c]
0	0.266	168	18.5	0.048	359	10.9	5.6
0.012	0.341	193	18.3	0.074	468	10.3	4.6
0.058	0.233	217	16.3	0.082	573	10.2	2.9
0.23	0.132	233	15.6	0.072	668	9.8	1.8
0.46	0.141	220	14.3	0.095	731	9.7	1.5

[a] All experiments contain an additional 5 μM pepsin.

[b] Deamidation measured from peptides identified in a proteomics search, configured to allow for deamidation as variable modification. Every peptide with a significant score was mined in the raw data for ion chromatograms of each peptide form (0-n deamidations, where n = maximum number of deamidations detected the database search. The ratio was determined using intensity-weighted ion chromatograms, where the intensities of all deamidated forms were normalized to the non-deamidated form. All analyses done using a custom module in Mass Spec Studio.

[c] Ratio of the deamidation ratio (antigenic) to the deamidation ratio (non-antigenic).
